# Bayesian data analysis reveals no preference for cardinal Tafel slopes in CO_2_ reduction electrocatalysis

**DOI:** 10.1038/s41467-021-20924-y

**Published:** 2021-01-29

**Authors:** Aditya M. Limaye, Joy S. Zeng, Adam P. Willard, Karthish Manthiram

**Affiliations:** 1 Department of Chemical Engineering, MIT, Cambridge, MA USA; 2 Department of Chemistry, MIT, Cambridge, MA USA

**Keywords:** Electrocatalysis, Scientific data, Statistics

## Abstract

The Tafel slope is a key parameter often quoted to characterize the efficacy of an electrochemical catalyst. In this paper, we develop a Bayesian data analysis approach to estimate the Tafel slope from experimentally-measured current-voltage data. Our approach obviates the human intervention required by current literature practice for Tafel estimation, and provides robust, distributional uncertainty estimates. Using synthetic data, we illustrate how data insufficiency can unknowingly influence current fitting approaches, and how our approach allays these concerns. We apply our approach to conduct a comprehensive re-analysis of data from the CO_2_ reduction literature. This analysis reveals no systematic preference for Tafel slopes to cluster around certain "cardinal values” (e.g. 60 or 120 mV/decade). We hypothesize several plausible physical explanations for this observation, and discuss the implications of our finding for mechanistic analysis in electrochemical kinetic investigations.

## Abstract (web summary)

The Tafel slope in electrochemical catalysis is usually determined from experimental data and remains error-prone. Here, the authors develop a Bayesian approach for Tafel slope quantification, and apply it to study the prevalence of certain "cardinal" Tafel slopes in the electrochemical CO_2_ reduction literature.

## Introduction

Modern tools in data science provide the capability to reduce or outright eliminate sources of human bias in the analysis and interpretation of experimental measurements. Despite the wide availability of these tools, many communities continue to rely on more primitive and bias-prone methods of data analysis. The calculation of Tafel slopes through linear least-squares fitting is one prominent example. Here, we present a robust Bayesian approach for analyzing electrochemical current–voltage measurements that (1) eliminates the need to manually exclude points in limiting-current regimes, and (2) provides a well-defined measure of uncertainty in the fitting parameters (e.g., the Tafel slope). By applying this approach to a large set of literature data, we identify a systematic but unjustified tendency for the assignment of Tafel slopes to specific “cardinal” values. This finding highlights the role that modern data science can play in uncovering and eliminating hidden sources of bias that exist within various scientific communities.

Current–voltage measurements are a fundamental characterization tool for electrochemical systems, as they report on the propensity for system response when pushed out of equilibrium by a thermodynamic driving force. In the context of electrochemical catalysis, current–voltage behavior is often summarized by the Tafel slope, a parameter that quantifies the amount of electrochemical driving force required to produce a logarithmic increase in the observed current^1^. Nearly all studies that develop a novel electrochemical catalyst report a Tafel slope, and it is considered an important figure of merit when comparing catalysts. In principle, the Tafel slope contains information about the microscopic mechanism underlying the operation of a catalyst. The elementary kinetic steps of idealized reaction mechanisms imply a strong tendency for Tafel slopes to exhibit certain “cardinal values”^2,3^, and these cardinal values are frequently referenced in the kinetic analysis of catalytic materials. However, several notable studies have reported Tafel slopes that differ significantly from their predicted cardinal values^4,5^. Because the kinetic steps associated with these cardinal values are ubiquitous, there is a tendency to interpret Tafel slopes with off-cardinal values as if they represent a truly cardinal value that has been altered by sources of experimental error, despite relatively incomplete error quantification^6–12^. The flaw in this interpretive strategy is that it relies on the validity of idealizing assumptions about the underlying kinetic mechanism, and does not account for the numerous ways in which deviations from ideality can influence the Tafel slope^13^.

Despite the scientific and engineering relevance of the Tafel slope, current literature approaches for estimating this parameter from measured current–voltage data require subjective human intervention, and are susceptible to numerous sources of systematic error. Subjective considerations in the fitting procedure (namely, the manual demarcation of a linear fit region) make it impossible to determine, in a truly unbiased manner, the intrinsic distribution of Tafel slopes, and whether they cluster around cardinal values. In addition, human interventions in the fitting procedure enable researcher bias, both inadvertent and intentional, to influence a quantitative catalyst benchmark. Such biases are difficult to recognize without re-examining primary source data.

To address these concerns, we advance an alternative Bayesian data analysis method that enables unbiased Tafel slope estimation. This method provides robust, distributional uncertainty quantification, elucidating the credible range of Tafel slope values consistent with the measured data. Because our method eliminates subjectivity in the fitting process, it enables us to fairly evaluate the prevalence of cardinal Tafel slopes within reanalyzed literature data.

In this paper, we begin by describing common literature practices for assigning Tafel slopes from experimental current–voltage data. Subsequently, we develop the mathematical formalism behind our Bayesian approach to Tafel slope estimation, and discuss its associated benefits compared to existing approaches. Using synthetic data, we illustrate the benefits of our approach, and show how it can be combined with iterative data acquisition procedures to systematically reduce uncertainty in Tafel slope estimates. Finally, we apply our approach to a large set of CO_2_ reduction catalyst data from the literature, and compare our Tafel slope estimates to the reported values. We found that clustering of reported Tafel slopes around cardinal values is unjustified, and likely reflects systemic bias across the field. We conclude by hypothesizing several plausible sources of mechanistic nonideality and estimating their ability to modify Tafel slopes from their cardinal values.

## Results

### Tafel slopes in electrocatalysis

Electrochemical systems operate by converting the energy stored in chemical bonds into electrical work (or vice versa) by means of electron transport through an external circuit. The electron transport process originates at an electrochemical interface, where a portion of the external circuit (an electrode) is contacted with a chemical system (a reactive electrolyte solution) in the presence of a catalyst. Catalyzed interfacial electron transfer serves to inextricably link the electrical dynamics of the external circuit to the chemical dynamics of the electrolyte. Electrochemical characterization techniques exploit this linkage, using the voltage and current measured in the external circuit to report on the thermodynamic driving forces and nonequilibrium currents in the chemical system. The simplest possible electrochemical experiment involves setting the applied potential at an electrode and measuring the resultant electrical current (or vice versa), generating a current–voltage trace.

Several phenomena can influence the shape of current–voltage traces. For example, the electronic properties of the electrode, the chemical identity of the catalyst material, the transport characteristics of reactive species in the electrolyte, and myriad other factors all play an important role in structuring current–voltage behavior^14^. When characterizing the performance of a catalyst material, we are most interested in the kinetic control regime of a current–voltage trace, where the measured current reports directly on the intrinsic rate of a chemical reaction at the interface. Under kinetic control, the current is generally expected to follow an exponential asymptotic dependence on the overpotential *η*, which quantifies the difference between the applied electrode potential and the equilibrium potential for the chemical reaction^1^. In the high ∣*η*∣ limit (specifically, ∣*η*∣ ≫ *k*_B_*T*/*e*, the thermal voltage), the logarithm of the kinetic current, *i*_kin_, should depend linearly on the applied potential. The (inverse) slope of this relationship is termed the Tafel slope,

1 $${\mathrm{Tafel}}\ {\mathrm{slope}}\equiv {\left.\frac{{d}\eta }{d{\mathrm{log}\,}_{10}{i}_{{\mathrm{kin}}}}\right|}_{| \eta | \gg {k}_{{\rm{B}}}T/e},$$

and is generally reported in units of mV/decade.

The Tafel slope is an important parameter to judge the performance of a catalyst because it quantifies the amount of additional applied potential required to observe a logarithmic increase in the measured current. Hence, studies that develop a novel electrocatalytic material often measure current–voltage data in the kinetic control regime, and then use these data to estimate a Tafel slope for their catalytic system. Experimental limitations impose a number of practical constraints on the Tafel slope estimation procedure. First, for several electrochemical reactions (CO_2_ reduction, N_2_ reduction, organic electrosynthesis, etc.), accurately determining a kinetic current for a specific reaction requires product quantification after a constant potential/current hold. Due to throughput limitations for quantification techniques, the Tafel slope often must be estimated from just a handful of data points (roughly, 3–10). Another important practical constraint arises from limiting-current phenomena observed in many electrocatalytic systems. At sufficiently high overpotentials, the reaction rate exceeds the rate of another physical process required for the reaction to proceed. In this regime, the system is no longer under kinetic control, and the measured current plateaus to a limiting current, becoming independent of the applied voltage^15^. Most commonly, limiting currents in CO_2_ reduction systems arise from diffusive transport limitations in the electrolyte, although several other physical reasons for plateau currents have been hypothesized and investigated in the literature^16–20^. Consequently, experimentally measured Tafel data usually starts out linear, but curves sublinearly at sufficiently high overpotentials.

**Fig. 1:**
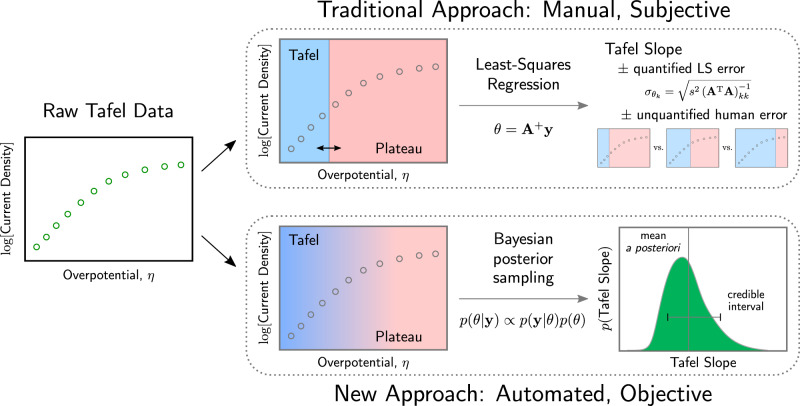
Schematic comparison of the traditional approach to Tafel fitting and the new approach we describe in this paper. Starting from raw current–voltage data on the left, the current literature approach begins with manual identification of a linear Tafel region on the plot. The rest of the data is discarded, and a linear fit to the Tafel region yields a Tafel slope, with an associated uncertainty corresponding to the standard error of the ordinary least-squares (OLS) estimator. In addition to this quantified uncertainty, an additional unquantified source of uncertainty arises from the manual selection of a Tafel region on the plot. The new approach described here considers all of the data in the context of a nonlinear model that smoothly interpolates between the traditional Tafel region and a plateaued region (e.g., due to mass transport limitations). Our approach uses a Monte Carlo method to sample from the Bayes posterior distribution over the parameters of the model, yielding a probabilistic distribution over Tafel slopes that are consistent with the measured data.

In the face of these practical limitations, studies in the literature use a relatively standard protocol for estimating the Tafel slope, depicted schematically in the upper half of Fig. 1. First, a researcher must manually identify an ad hoc cutoff between a linear, kinetically controlled regime (the Tafel regime) and a limiting-current (plateau) regime^21^. All data points in the plateau regime are subsequently discarded, and a Tafel slope is fitted by ordinary least-squares (OLS) linear regression to the data in the Tafel regime. The OLS procedure offers a prescription for extracting the standard error of the Tafel slope; this standard error is sometimes used to construct a confidence interval for the Tafel slope estimate^22,23^.

We believe the current standard literature practice bears several drawbacks. First and foremost, manual identification of a cutoff between the kinetic and limiting-current regimes introduces subjectivity into Tafel slope estimation, potentially incorporating undesirable human influence into the quantification of an important metric for electrocatalyst performance. Second, reporting the error associated with a linear fit to a manually selected set of data points systematically underestimates the actual error associated with estimating a Tafel slope from a small number of data points. The OLS slope standard error quantifies the uncertainty associated with the linear fit to a given set of data, but there is an additional unquantified error associated with selection of the linear regime. Third and finally, ad hoc selection of a regime cutoff can introduce a systematic bias in the Tafel slope, since the final few points of the kinetic regime will suffer at least some effects from limiting-current curvature, causing the current at these points to deviate slightly from the true kinetic current.

### Bayesian data analysis algorithm

Our approach for Tafel slope estimation seeks to obviate manual demarcation between the linear and plateau regions in current–voltage data. To this end, we choose to fit all current–voltage data measured in a Tafel experiment to a phenomenological model that smoothly interpolates between the kinetic control and plateau regimes. The model reads,

2 $$\frac{1}{i(\eta )}=\frac{1}{{i}_{{\rm{lim}}}}+\frac{1}{{i}_{0}\exp \left[{m}_{{\rm{T}}}^{-1} | \eta | \right]},$$

where *i*(*η*) is the measured current density as a function of overpotential. The unknown parameters in the model are ${i}_{{\rm{lim}}}$, the limiting-current density, *i*_0_, the exchange current density, and ${m}_{{\rm{T}}}^{-1}$, the inverse Tafel slope. The mathematical structure of Eq. (2) can be shown to arise, for example, when the surface concentration of a redox-active species changes with applied overpotential due to diffusive transport effects. Alternatively, Eq. (2) could be motivated by interpreting the presence of limiting-current phenomena as an additional series resistance to current in the equivalent circuit for an electrochemical cell^1^. Most generally, this model can describe any physical phenomenon that imposes a “speed limit” on the passed current^24^.

Typically, the parameters in a model like Eq. (2) would be adjusted to achieve optimal agreement between the model and experimental data. Despite the nonlinearity inherent to the model, numerical optimization schemes for determining the optimal set of parameters are mature and well-studied. Here, we employ a different approach based on Bayesian sampling that can quantify not only an optimal set of parameters but also a distribution over the likely values of the model parameters given the available experimental data^25^. Before carrying out any current–voltage measurements, we generally have at least some idea of the reasonable values of parameters in Eq. (2). For example, one would be very leery of a Tafel slope *m*_T_ ∉ [10^1^, 10^3^] mV/decade, and one can use tabulated values of a species diffusion coefficient and an estimate of the cell boundary layer thickness to compute a ballpark estimate of a limiting current, ${i}_{{\rm{lim}}}$, arising from diffusive transport limitations^1,24^. For a general set of parameters *θ*, this knowledge is encoded in a prior distribution over the parameters *p*(*θ*). Upon observing some data **y**, we can compute an updated posterior distribution *p*(*θ*∣**y**) (the probability of the parameters given the observed data) using Bayes’ rule^26^,

3 $$p(\theta | {\bf{y}})=\frac{p({\bf{y}}| \theta )\times p(\theta )}{p({\bf{y}})}.$$

The likelihood *p*(**y**∣*θ*) is supplied by a model like Eq. (2), in conjunction with an assumption about the distribution over the error at each data point. Here, we assume that the error at each data point is independently normally distributed with a standard deviation *σ* = 0.10 log units, although our approach is flexible in this respect (see Supplementary information for sensitivity analysis with respect to the *σ* parameter).

Despite the fact that the partition function *p*(**y**) is an unknown constant, Eq. (3) yields a prescription for sampling from the posterior distribution over parameters *p*(*θ*∣**y**). Briefly, one can use a Markov chain Monte Carlo sampling scheme^27^ to glean several parameter samples from the posterior distribution, which are expected to concentrate in density around the optimal values of the parameters (additional detail on implementation can be found in the Supplementary information). As depicted in the lower panel of Fig. 1, this Bayesian posterior sampling technique reveals both the optimal values of the model parameters and a distributional uncertainty estimate over their values, accounting for all sources of uncertainty in the model. If we truly want to collapse this distributional information to a single value of the parameter, say for quoting a Tafel slope associated with a catalyst, we can compute,

4 $$\langle \theta \rangle \equiv \int {d}\theta \cdot \theta \cdot p(\theta | {\bf{y}}),$$

where 〈*θ*〉 is termed the mean a posteriori (MAP) parameter estimate. When the posterior distribution *p*(*θ*∣**y**) is strongly peaked around an optimal set of parameters, the MAP estimate will line up with the parameters gleaned from a nonlinear optimization technique. When the posterior distribution is broad or multimodal, the MAP estimate and the optimal parameter values may differ, signaling a high degree of uncertainty associated with the optimal parameter estimate.

The Bayesian posterior sampling approach offers several key advantages over the traditional approach to Tafel slope estimation. First, it removes subjectivity from the analysis of Tafel data: users of our algorithm need to only select a model such as Eq. (2) to interpret the observed data, which can be justified on the basis of rigorous physical arguments, unlike a subjective delineation between linear and plateau regimes. Second, our approach yields accurate quantification of the uncertainty associated with a Tafel slope estimate. Specifically, we believe the distributional uncertainty quantification afforded by our algorithm will be useful when assessing and discriminating between disparate sets of experimental data. Finally, because the model in Eq. (2) analytically extrapolates away curvature-related attenuation of the kinetic current, our approach is free of the systematic bias present in current literature practice.

As a caveat, we stress that while the model in Eq. (2) is appropriate for fitting some current–voltage data in the CO_2_ reduction literature, experimental and theoretical studies have noted the possibility of multiple kinetic control regimes with distinct Tafel slopes before a limiting-current plateau regime^3,28,29^. This phenomenon can arise due to potential-dependent surface coverage effects or a potential-dependent switch in the microscopic reaction mechanism. When multiple distinct Tafel regimes are present, the experimental data must be interpreted under a model that allows for this possibility; once a suitable model is selected, the Bayesian posterior sampling approach can still be employed (see Supplementary information for additional discussion on model flexibility).

### Identifying and addressing data insufficiency

As mentioned previously, practical throughput considerations imposed by product quantification and other experimental requirements limit the amount of data generally used in a Tafel analysis to 3–10 points. In a survey of Tafel data reported in CO_2_ reduction studies in the literature, we found that a significant number of papers conduct Tafel analyses on a set of 3–5 data points within a narrow overpotential window. With such few data points, trends often appear linear, and so many studies simply extract the Tafel slope from a linear fit to all the data. While seemingly benign, estimating a Tafel slope without accounting for the possibility of limiting-current nonlinearity can lead to systematic error arising from data insufficiency. This phenomenon is elegantly identified and addressed by our Bayesian posterior sampling approach. For the sake of clarity, we illustrate how this systematic error can emerge by analyzing a set of synthetic data. The parable narrated by these data is easily relatable to a specific set of experimental data, and it emphasizes another distinct advantage of the Bayesian posterior sampling approach.

**Fig. 2:**
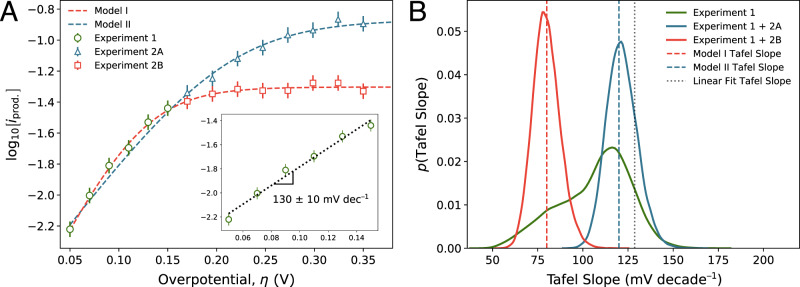
Bimodal posterior distributions signify Tafel slope ambiguity that cannot be clarified by the available data. **A** (inset) Synthetic current–voltage data collected in hypothetical Experiment 1 appears linear over a 100 mV overpotential region, with a Tafel slope of 130 ± 10 mV/decade. **A** Synthetic current–voltage data over a broader range of overpotentials. The dashed lines show two models (I, II) with different Tafel slopes and plateau currents that could both reasonably fit the Experiment 1 data. Synthetic error bars represent one standard error of the mean of uncertainty in the synthetic data. Experiment 2A (blue triangles) and 2B (red squares) represent two possible outcomes of experiments that probe a broader range of overpotentials, which can clearly distinguish between Model I and Model II. **B** Bayes posterior distributions over the Tafel slope determined by our algorithm given various sets of observed data. If the algorithm is fed just the Experiment 1 data, the posterior distribution over the Tafel slope is broad and weakly bimodal, indicating that the Experiment 1 data is insufficient to discriminate between Model I and Model II. When fit to the Experiment 2A or 2B data in addition to the Experiment 1 data, this bimodality splits cleanly into two separate modes centered at the Model I and Model II Tafel slopes. Note that the Tafel slope extracted from a linear fit to just the Experiment 1 data is distinct from both the Model I and Model II Tafel slopes.

The inset of Fig. 2A depicts a set of (synthetic) Tafel data measured over a 100 mV overpotential window. To the eye, the data looks entirely linear, and fitting a Tafel slope to the entire dataset using OLS regression yields a Tafel slope of 130 mV/decade, with a standard error of 10 mV/decade. However, Fig. 2A illustrates the issue with this traditional Tafel analysis. Indeed, the original set of green data in the narrow overpotential regime is essentially entirely consistent, within experimental error, with two models possessing very different Tafel slopes. Model I has a Tafel slope of 80 mV/decade, while Model II has a Tafel slope of 120 mV/decade. Despite this wide berth in Tafel slope, the models align in the initial overpotential window because of their distinct limiting currents ${i}_{{\rm{lim}}}$, which differ by a modest half order of magnitude. Clearly, the set of data measured in the inset of Fig. 2A is insufficient to distinguish between the two models, but these data insufficiency is entirely hidden by the traditional analysis approach.

Unlike the traditional analysis approach, Bayesian posterior sampling correctly identifies the Tafel slope ambiguity present in the original data. The green trace in Fig. 2B depicts the posterior distribution over the Tafel slope using solely the green data. This distribution is broad and markedly multimodal, with concentrations of probability density around the Model I and II Tafel slopes. In this manner, Bayesian posterior sampling correctly surmises that the original data are insufficient to pin down a value of the Tafel slope with high certainty. One possible solution to this issue is to measure additional data over a wider range of overpotentials. Depending on the true parameters of the electrochemical system under measurement, this experiment could yield either the blue data or the red data in Fig. 2A. When the Bayes posterior sampling algorithm is fed a combination of the green data and the red data, it correctly predicts a posterior distribution of Tafel slopes concentrated around the Model I Tafel slope. Conversely, when fed a combination of the green data and the blue data, it correctly predicts a posterior distribution of Tafel slopes concentrated around the Model II Tafel slope. In other words, multimodality in the posterior distribution predicted by our algorithm is a hallmark of data insufficiency; when the underlying insufficiency is addressed, the algorithm neatly splits the distributional modes according to the observed data.

We highlight a couple of important conclusions from the synthetic data analysis presented in Fig. 2. First, current–voltage data used for Tafel slope estimation should ideally be measured until clear curvature is observed. If such a measurement is unnecessarily inconvenient or impossible, one should attempt to quantify the limiting current either through back-of-the-envelope estimates or through direct experimental control over the limiting current (e.g., with a rotating-disk electrode), and ensure that data used to estimate Tafel slopes is collected well below the limiting-current density. Without information that elucidates the magnitude of the limiting current, it is impossible to ascertain the degree of limiting-current-induced attenuation suffered by the current measured in the Tafel regime. Consequently, Tafel slopes estimated on the basis of a linear Tafel plot measured in a small overpotential window are likely systematically unreliable, and can harbor significant unquantified uncertainty. Second, the synthetic data analysis illustrates how the Bayesian posterior sampling approach can be employed iteratively with data acquisition efforts. Since the posterior distributions accurately quantify the uncertainty associated with a Tafel slope estimated given available data, an experimentalist can use this uncertainty information to guide future data acquisition until a desirable uncertainty threshold is achieved.

### Evaluating cardinal preferences in literature data

In addition to being an important metric in assessing catalyst performance, the Tafel slope can be valuable because it may yield insight into the mechanism of a catalyzed electrochemical reaction. The connection between the Tafel slope, a macroscopically measurable quantity, and the microscopic reaction mechanism is derived using microkinetic analysis invoking a whole host of ideality assumptions^2,3^. For an electrochemical reaction that proceeds through a number of elementary steps, one must assume that a single step determines the rate, and that all steps prior to the rate-determining step (RDS) are in quasi-equilibrium. Each of the quasi-equilibrated elementary steps carries an associated equilibrium constant, which is possibly dependent on the applied potential. For potential-dependent equilibrium constants, one must additionally assume that the potential dependence goes exponentially in the (strictly integer) number of electrons transferred in the elementary step. The RDS has an associated forward rate constant, which is assumed to have a Butler–Volmer-like dependence on the applied potential, with a symmetry coefficient *α* = 1/2^1^. Under these restrictive assumptions, one can derive (see Supplementary information) an equation for the Tafel slope of the entire chemical reaction (at *T* = 298 K),

5 $${\rm{Tafel}}\ {\rm{slope}}=\frac{60\, {\mathrm{mV}}/{\rm{decade}}}{n+q/2},$$

where *n* is the total number of electrons transferred in elementary steps prior to the RDS, and *q* is the number of electrons transferred in the RDS.

Equation (5) gives rise to the so-called “cardinal values” of the Tafel slope, which arise from evaluating the Tafel slope for different values of (*n*, *q*). Tafel slope values of 120, 60, and 40 mV/decade are familiar to most electrochemists, and arise from (*n*, *q*) = (0, 1), (1, 0), and (1, 1), respectively. Researchers routinely appeal to cardinal values to extract microscopic insight from experimentally measured Tafel slopes. A common argumentative thrust goes: “my catalyst has a Tafel slope of 110 mV/decade, which is reasonably close to 120 mV/decade, indicating that the reaction proceeds through a rate-limiting first electron transfer step.” This line of reasoning is only truly valid if the typical catalyst satisfies the ideality assumptions involved in deriving Eq. (5), a point that has been emphasized several times in the literature^3–5^. Comprehensive analysis of literature Tafel data can shed light on whether a typical catalyst satisfies these strict assumptions; if this is indeed true, then literature Tafel slopes should tend to cluster around the cardinal Tafel values predicted by Eq. (5).

Our Bayesian posterior sampling algorithm for Tafel slope fitting allows us to carry out an unbiased, automated survey of literature Tafel data to quantitatively test whether Tafel slope values reported in the literature show any preference for cardinal values. In this study, we choose to focus on reanalyzing Tafel data from the CO_2_ reduction literature. We focus on this subsection of the literature because CO_2_ reduction is a burgeoning field with diverse catalyst materials and morphologies^30^, and because product quantification requirements place Tafel analysis in this field in the low-data regime, as discussed previously. To carry out the literature survey, we digitized 344 distinct Tafel datasets from the CO_2_ reduction literature and fed the resultant data to the Bayesian posterior sampling algorithm to produce reanalyzed estimates of the Tafel slope. Further information on the data mining and analysis procedure can be found in the “Methods” section.

Our re-analysis procedure uses Eq. (2) to interpret the literature Tafel datasets. As mentioned earlier, this model is only truly appropriate in the case of one kinetic control regime associated with a single Tafel slope; it cannot accurately capture current–voltage behavior under multiple kinetic regimes, which may be operative in at least some of the datasets we have analyzed. However, absent independent experimental confirmation of the physical mechanism underlying the observed multiple kinetic regimes (e.g., from spectroscopy or surface imaging), it is difficult to rigorously select a single model from the plethora that arise from enumerating microkinetic possibilities for intermediates in CO_2_ reduction. Since the papers from the literature that we have reanalyzed fit a single Tafel slope to their data, and because they lack the experimental evidence required to pin down a richer physical model describing their data, we believe that uniform application of the model in Eq. (2) is an appropriate choice for our literature survey study. Our usage of Eq. (2) to interpret the data should not be construed as a blanket endorsement of this model in Tafel analysis. Indeed, if there is solid experimental evidence motivating the usage of a different kinetic model for a specific CO_2_ reduction catalyst system, it can and should be employed under our Bayesian framework.

**Fig. 3:**
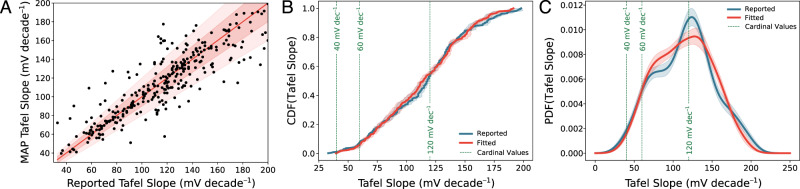
Unbiased refit of literature data using our Bayesian analysis approach reveals little preference for cardinal values of the Tafel slope for CO_2_ reduction catalysts. **A** Correlation plot of reported Tafel slopes from the literature against MAP Tafel slopes fitted by our algorithm on identical data. The solid red line represents a perfect agreement, while the red filled intervals are lines representing 10% and 20% relative error. **B** Cumulative distribution function of the Tafel slopes reported in literature data (blue), and those refitted by our algorithm (red). Error intervals correspond to one standard deviation of bootstrapped resamples. **C** Kernel density estimates (KDEs) of the empirical probability distribution function of Tafel slopes reported in literature data (blue) and MAP Tafel slopes refitted by our algorithm (red). Error intervals correspond to one standard deviation of bootstrapped resamples. Green dashed lines in both (**B**, **C**) correspond to cardinal values of the Tafel slope predicted by Eq. (5).

Figure 3A depicts a correlation plot of the MAP Tafel slope estimated by the Bayes posterior sampling approach versus the literature-reported Tafel slope. A significant fraction of the datasets fall within the 20% parity line, a strong sign that our algorithm produces Tafel slopes that are consistent with literature values when seeing identical data. In addition, the MAP Tafel slope does not seem to systematically overestimate or underestimate the literature-reported value over a wide range of reported Tafel slopes. We note that complete parity between the MAP and literature Tafel slopes should not be expected; as explained previously, due to the possibility for subjectivity and systematic error with current literature practice for Tafel estimation, the MAP estimates derived by our algorithm are arguably more trustworthy than the literature-reported values.

Figure 3B, C depicts estimates of the distributional tendencies of the MAP and literature-reported Tafel slopes. Figure 3B plots the empirical cumulative distribution function (CDF) of the MAP Tafel slope (red trace) and the literature-reported Tafel slopes (blue trace). The low-opacity intervals in both Fig. 3B, C span one standard deviation of several bootstrapped resamples drawn with replacement (see “Methods” section for additional detail on the bootstrapping procedure), and are useful for examining the sensitivity of our distributional results to the specific subsampling of literature data we have chosen to analyze^31^. The CDF value for a given Tafel slope value *m*_T_ tallies the running fraction of datasets that have a Tafel slope value of at most *m*_T_. If Tafel slopes truly cluster around cardinal values, the running fraction should increase sharply around those preferred values, and one would expect to see sigmoidal features in the CDF at the cardinal values. Figure 3 shows little evidence of such locally sigmoidal behavior; rather, we see something resembling a straight line, corresponding to a roughly uniform distribution over the range of Tafel slopes considered.

We can visualize the distributional data in a different way by examining the empirical probability distribution function (PDF) of the Tafel slopes. Estimating the PDF of a distribution given a set of samples is a notoriously difficult problem in statistics because relatively small amounts of sampling noise can result in the presence of spurious peaks in the PDF. Several techniques exist for tackling this problem; here, we employ Gaussian kernel density estimation, which constructs an estimate of the PDF by summing appropriately normalized Gaussian kernel functions centered at each of the observed data points. Additional details on the kernel density estimation procedure are reported in the “Methods” section. Figure 3C shows a kernel density estimate of the PDF of the MAP (red trace) and literature-reported (blue trace) Tafel slopes. Based on the shape of the PDFs and their corresponding standard errors, we conclude that there is a slight preference to assign Tafel slope values around 70 and 125 mV/decade in the literature that essentially disappears when the same data are reanalyzed with our approach. While a small peak persists ~125 m /decade in the MAP Tafel slopes, the height of the peak is roughly within the error bound. We also note that any residual preference for cardinal values in the MAP Tafel slopes can possibly be explained by data acquisition biases. While the Bayesian approach we develop removes subjectivity from data analysis, we cannot remove biases introduced during data collection; it is at least plausible that such biases exist given that the literature-reported values significantly overestimate the concentration of Tafel slopes ~120 mV/decade compared to the MAP values.

The results presented in Fig. 3 combine data from several studies using different catalyst materials to assess whether or not a preference for cardinal Tafel slopes exist broadly across all CO_2_ reduction catalysts. In order to confirm that this apparent lack of cardinal preference in the entire dataset is not simply an artifact introduced by pooling together separate catalyst materials, which each individually exhibit a cardinal preference, we broke out the PDF analysis in Fig. 3 according to catalyst material identity. Upon examining the results from the breakout analysis (see Supplementary information), we conclude that the apparent lack of cardinality we find in Fig. 3 indeed persists when separately examining Tafel slopes from common materials used in CO_2_ reduction catalysts: Ag, Au, Cu, Sn, and Zn. Curiously, it appears that Bi-based materials do show a preference for Tafel slope values ~120 mV/decade, which may inform future mechanistic studies on these catalysts. As a caveat, we note our Bi results comprise only 27 distinct Tafel datasets, and are hence subject to a high degree of variability arising from the specific set of studies we chose to reanalyze; future work that attempts to weigh in on this question should ideally perform new experiments on well-controlled Bi surfaces and use the data acquisition and analysis recommendations identified in this work. Taken together, the results presented in Fig. 3 and the material breakout analysis in the Supplementary information lead us to conclude that, when analyzed in an unbiased fashion, experimental data in the literature does not support a systematic preference for cardinal values of the Tafel slope among CO_2_ reduction catalysts.

While we are not in a position to identify the cause for deviation from cardinal Tafel slope values in each specific Tafel dataset comprising Fig. 3, we advance the hypothesis that these deviations could originate from physical nonidealities that violate the assumptions involved in deriving Eq. (5). To test this hypothesis, we attempt to ascertain the manner in which a select few simple physical nonidealities can adjust the PDF of Tafel slopes that would otherwise be concentrated around cardinal values. In this regard, we consider three possible physical effects that violate the ideality assumptions used to arrive at Eq. (5). These three effects comprise a very small subset of the menagerie of physical nonidealities that could operate in CO_2_ reduction electrocatalysis; our goal is simply to show that these effects can spoil a preference for cardinal values of the Tafel slope, not to single out these particular effects as the only nonidealities present in CO_2_ reduction.

First, we consider the possibility that the symmetry coefficient *α* ≠ 1/2. Such deviations could arise, for example, due to disparate local slopes of the Marcus free energy surfaces at their crossing point, or reactant species position fluctuations in the electrochemical double layer^32–35^. Second, we examine the effect of partial charge transfer or surface dipole formation in the adsorption of CO_2_ to the electrode surface, phenomena that have been hypothesized and characterized in prior studies on CO_2_ reduction^29,36^. Mathematically, the formation of a surface dipole introduces a potential dependence in the adsorption equilibrium constant, which mathematically resembles a partial charge transfer parameter *γ*. Third, we introduce a possible Frumkin correction *f* originating from the protrusion of an electrode-adsorbed species into the electrochemical double layer, which attenuates the applied potential due to electrostatic screening effects^1^. The Frumkin correction is most important at low supporting salt concentration (and hence large resultant electrolyte screening length), but it has been considered in the context of CO_2_ reduction electrocatalysis^29^. Each of these nonidealities depends on the value of a nonideality parameter. Of course, we have no idea, at least a priori, about the distribution of values these nonideality parameters can take in typical CO_2_ reduction catalyst systems. In the absence of information, we make the maximally ignorant choice, and assume that the nonideality parameters are drawn from uniform distributions within a reasonable set of bounds. Once we postulate these uniform distributions, we can examine how randomly selected nonideality parameters deform a distribution of Tafel slopes that begins concentrated around cardinal values.

**Fig. 4:**
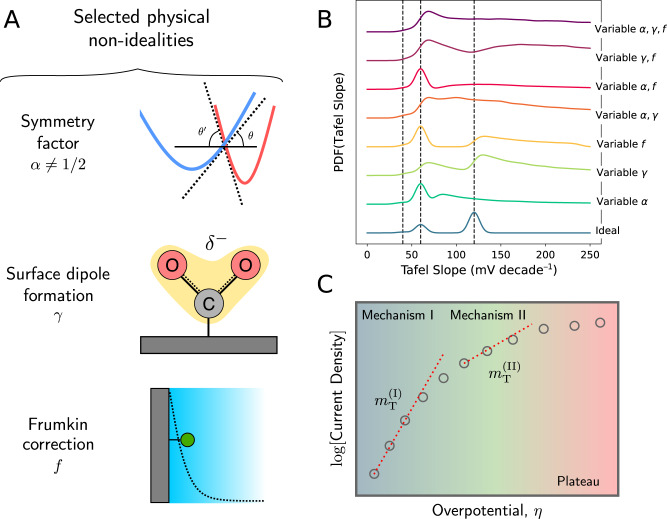
Physical hypotheses for the lack of observed cardinality in literature Tafel slopes. **A** Schematic of three selected physical nonidealities that can affect the measured Tafel slope. **B** (blue trace) Synthetic kernel density estimate of the probability distribution over Tafel slopes for a random CO_2_ reduction catalyst, peaked around the cardinal values predicted by Eq. (5). **B** (other traces) Several synthetic kernel density estimates of the probability distributions over the Tafel slope generated from including random values of different parameters governing physical nonidealities. **C** Schematic illustrating the possibility of measuring data across separate kinetic regimes in a Tafel analysis. Due to a switch in mechanism, different overpotential regimes exhibit different Tafel slopes, complicating interpretation of a single Tafel slope value fit straddling both regimes.

The blue trace in Fig. 4B shows a distribution of Tafel slopes concentrated around the cardinal values predicted by Eq. (5). The relative heights of the cardinal value peaks are selected by artificially binning the distribution over MAP Tafel slopes into buckets centered around the cardinal values. The effects of randomly drawn physical nonidealities on this distribution can be examined using Monte Carlo simulation. Briefly, we sample a Tafel slope from the distribution depicted in the blue trace, sample the values of one or more nonideality parameters, and finally calculate the resultant Tafel slope in the presence of nonidealities (see “Methods” section for additional details). Repeating the sampling procedure several times yields distributions over the Tafel slope, depicted as multicolored traces in Fig. 4B for different sets of nonidealities. Evidently, even rather mundane pieces of additional physics like the ones discussed above can produce stark changes in the distribution of Tafel slopes, spanning a range of behavior from moving certain peaks away from cardinal values to smearing out the entire distribution. While we cannot prove with certainty that physical nonidealities are responsible for the lack of observed Tafel cardinality in the literature, the results in Fig. 4B demonstrate that this is at least a plausible explanation for the observed behavior.

Alternatively, the observed lack of cardinality may also be a consequence of interpreting current–voltage data measured under several disparate kinetic regimes through the lens of Eq. (2), which cannot capture these intricacies. As illustrated schematically in Fig. 4C, the kinetic regimes may exhibit different Tafel slopes; in this case, mechanistic interpretation of a single Tafel slope extracted by fitting Eq. (2) to the data is inappropriate. Indeed, as examined in more detail in the Supplementary information, fitting synthetic data generated from a model with multiple cardinal Tafel slope regimes using Eq. (2) can produce an off-cardinal Tafel slope value. This underscores the need to rigorously characterize the several physical complexities present in catalytic systems for CO_2_ reduction, as they can complicate mechanistic interpretation guided solely by the Tafel slope.

Taken in their entirety, the results presented in Figs. 3 and 4 present some compelling reasons for the community to rethink the current approach of deducing mechanistic information purely from Tafel slope data. Indeed, the prevalence of Tafel slopes in the CO_2_ reduction literature that do not fall neatly on cardinal values suggests that physical nonidealities, omitted by Eq. (5), may be commonplace in typical catalytic systems. The familiar approach of “rounding” experimentally measured Tafel slopes to their nearest cardinal value to guide mechanistic interpretation, then, leaves one prone to interpreting experimental data in an overly simplistic manner. Rather than hand-wave away the physical complexities present in catalytic systems with strong ideality assumptions, we believe it is important to interpret Tafel data alongside several other pieces of experimental data (e.g., more diverse electrochemical kinetic data, surface-sensitive spectroscopy, materials characterization, etc.). In this manner, one can take a non-cardinal Tafel slope (and its associated uncertainty based on the data) at face value, and build a holistic physical picture that attempts to explain the deviation from cardinality in a manner consistent with all other experimental observations. Ideally, all these observations can be interpreted in the context of a richer model that allows one to determine the true breadth of physical phenomena present across a wide range of operating parameters, as has been done in some select studies in the literature^2,28,37^. We believe our Bayesian data analysis approach will be equally useful for rigorously quantifying parametric uncertainties in the suggested new paradigm for kinetic data interpretation.

## Methods

### Data mining and reanalysis

We built up a dataset of Tafel measurements reported in the literature by manually extracting figures from published papers and digitizing them using the WebPlotDigitizer tool.^38^ A full accounting of all papers and corresponding figures can be found in the Supplementary information. When selecting datasets to analyze, we excluded those that reported continuous current–voltage data, because it is difficult to ascertain the underlying data density associated with a continuous curve, because our method is meant to address the unique challenges of estimating Tafel slopes with a small amount of data, and because continuous current–voltage data may be unreliable because product selectivity is not always 100%, especially in CO_2_ reduction. We also excluded datasets that reported current–voltage data but did not report an explicit value of the Tafel slope. We assumed that all datasets were collected with appropriate experimental techniques (IR correction for solution resistance has already been applied, etc.), and did not modify or omit any data from a figure during the digitization process. After digitization, each dataset was tagged with manually entered metadata to facilitate reanalysis. A full accounting of the metadata fields, as well as a complete record of all scraped data and metadata, is available in the Supplementary information.

Reanalysis of the data was carried out using the Python-based julius package, developed in-house to handle data collation and Bayesian posterior sampling workflows. In order to determine prior distributions for the parameters, we first find an optimal set of parameters θ^*^ for the limiting-current model using an implementation of the trust-region reflective algorithm implemented in the optimization and root finding package included in SciPy^39^. For each model parameter *θ*_*i*_, we select uniform prior distributions supported on the interval $[0,a\cdot {\theta }_{i}^{* }]$, with *a* = 10 (note that all parameters we fit in this study are strictly nonnegative). For all models studied here, we find that the posterior distribution does not depend on the value of *a*, indicating that the data imposes strong preferences on the optimal model fit (see Supplementary information for detailed sensitivity analysis). For each dataset, we draw *N* = 4 × 10^4^ total samples (10^4^ samples from four independent chains, each burning their first 2000 samples) from the posterior distribution using the No-U-Turn Hamiltonian Monte Carlo sampler implemented in the PyMC3 probabilistic programming package^40^.

In total, we reanalyzed 344 distinct Tafel datasets. Figure 3A restricts to both reported and MAP Tafel slopes *m*_T_ ∈ [0, 200], which comprises 300 distinct Tafel datasets. A correlation plot including all analyzed Tafel datasets is reported in the Supplementary information.

### Kernel density estimation

We use kernel density estimation (KDE) to estimate probability distributions given a finite set of samples. KDEs are used in the distributional visualizations in Figs. 2, 3, and 4. We use the Gaussian KDE function in the statistics package included in SciPy, and use Scott’s rule for bandwidth selection in Figs. 2 and 3. Since the estimates in Fig. 4 are meant to emulate the result of a single simulated experimental observation with some associated error, here we use a pre-specified bandwidth of 6 mV/decade.

### Bootstrap resampling

We carry out a bootstrap resampling procedure to quantify the degree of variability of the results in Fig. 3 associated with our choice of a specific subset of literature data^31^. Essentially, we posit that the observed distribution over the Tafel slope is a good estimate of the true underlying distribution, and then resample several datasets of the same size as the original dataset from this distribution with replacement (i.e., samples can show up more than once, or not at all). The error intervals presented in Fig. 3A, B are gleaned from one standard deviation of 20 such bootstrapped resamples.

### Monte Carlo simulation

We use Monte Carlo simulation to estimate the distributional changes precipitated in a Tafel slope distribution by the physical nonidealities identified in the main text. To carry out this procedure, we begin with the distribution presented in Fig. 4A, which is generated by artificially bucketing the MAP Tafel slopes from the literature analysis into the bins {[0, 50), [50, 90), [90, *∞*)}. We sample the physical nonideality parameters according to *α* ~ Unif[0.20, 0.80], *γ* ~ Unif[0, 1], *f* ~ Unif[0.50, 1.00], where Unif[*a*, *b*] signifies a uniform distribution supported on the interval [*a*, *b*]. For each set of nonidealities, we draw *N* = 4 × 10^4^ total posterior samples (10^4^ samples from four independent chains, each burning their first 500 samples). The equations governing modifications to the Tafel slope based on physical nonidealities are worked out in the Supplementary information. A sensitivity analysis of the Tafel slope distributions including nonidealities with respect to the bounds of the uniform distributions over nonideality parameters is also reported in the Supplementary information. All Monte Carlo simulation is again carried out using the PyMC3 probabilistic programming package^40^.

## Supplementary information

Supplementary Information

Peer Review File

## Data Availability

Data that supports the findings of this study is available under CC BY 4.0 (https://creativecommons.org/licenses/by/4.0/) in Zenodo (10.5281/zenodo.3995021), with the exception of the excerpted figures from other articles as described in the Supporting information. The excerpted figures are reused under an agreement between MIT and the publishers of the articles (https://libraries.mit.edu/scholarly/publishing/using-published-figures/), where the copyright is owned by the publishers.

## Code availability

Code that supports the findings of this study is available under the MIT License (https://opensource.org/licenses/MIT) in Zenodo (10.5281/zenodo.3995021).

## References

[1] Bard, A. J., Faulkner, L. R., Leddy, J. & Zoski, C. G. *Electrochemical Methods: Fundamentals and Applications*, Vol. 2 (Wiley, New York, 1980).

[2] Marshall AT (2018). Using microkinetic models to understand electrocatalytic reactions. Curr. Opin. Electrochem..

[3] Shinagawa, T., Garcia-Esparza, A. T. & Takanabe, K. Insight on Tafel slopes from a microkinetic analysis of aqueous electrocatalysis for energy conversion. *Sci. Rep*. **5**, 13801 (2015). 10.1038/srep13801 PMC4642571 26348156

[4] Fang Y-H, Liu Z-P (2014). Tafel kinetics of electrocatalytic reactions: from experiment to first-principles. ACS Catal..

[5] Dunwell M, Luc W, Yan Y, Jiao F, Xu B (2018). Understanding surface-mediated electrochemical reactions: CO_2_ reduction and beyond. ACS Catal..

[6] Gattrell M, Gupta N, Co A (2006). A review of the aqueous electrochemical reduction of CO2 to hydrocarbons at copper. J. Electroanal. Chem..

[7] Chang X (2018). Tuning Cu/Cu2O interfaces for the reduction of carbon dioxide to methanol in aqueous solutions. Angew. Chem..

[8] Zhang L, Zhao Z-J, Gong J (2017). Nanostructured materials for heterogeneous electrocatalytic CO2 reduction and their related reaction mechanisms. Angew. Chem. Int. Ed..

[9] Lee CW, Cho NH, Yang KD, Nam KT (2017). Reaction mechanisms of the electrochemical conversion of carbon dioxide to formic acid on tin oxide electrodes. ChemElectroChem.

[10] Lu Q, Rosen J, Jiao F (2015). Nanostructured metallic electrocatalysts for carbon dioxide reduction. ChemCatChem.

[11] Lee CW (2018). New challenges of electrokinetic studies in investigating the reaction mechanism of electrochemical CO2 reduction. J. Mater. Chem. A.

[12] Corbin N, Zeng J, Williams K, Manthiram K (2019). Heterogeneous molecular catalysts for electrocatalytic CO2 reduction. Nano Res..

[13] Lu Q, Jiao F (2016). Electrochemical CO2 reduction: electrocatalyst, reaction mechanism, and process engineering. Nano Energy.

[14] Schmickler, W. & Santos, E. *Interfacial Electrochemistry* (Springer Science & Business Media, 2010).

[15] Clark EL (2018). Standards and protocols for data acquisition and reporting for studies of the electrochemical reduction of carbon dioxide. ACS Catal..

[16] Williams K (2019). Protecting effect of mass transport during electrochemical reduction of oxygenated carbon dioxide feedstocks. Sustain. Energy Fuels.

[17] Singh MR, Clark EL, Bell AT (2015). Effects of electrolyte, catalyst, and membrane composition and operating conditions on the performance of solar-driven electrochemical reduction of carbon dioxide. Phys. Chem. Chem. Phys..

[18] Singh MR, Goodpaster JD, Weber AZ, Head-Gordon M, Bell AT (2017). Mechanistic insights into electrochemical reduction of CO2 over Ag using density functional theory and transport models. Proc. Natl Acad. Sci. USA.

[19] Zhang BA, Ozel T, Elias JS, Costentin C, Nocera DG (2019). Interplay of homogeneous reactions, mass transport, and kinetics in determining selectivity of the reduction of CO2 on Gold electrodes. ACS Cent. Sci..

[20] Brown, S. M. *Catalysis and Reactor Engineering for the Electrochemical Conversion of Carbon Dioxide to Carbon Monoxide*. Ph.D. thesis, Massachusetts Institute of Technology (2019).

[21] Voiry, D. et al. Best practices for reporting electrocatalytic performance of nanomaterials. *ACS Nano* **12**, 9635–9638 (2018). 10.1021/acsnano.8b07700 30347985

[22] Rosenfeld, M. J. OLS in matrix form. https://web.stanford.edu/mrosenfe/soc_meth_proj3/matrix_OLS_NYU_notes.pdf (2013).

[23] Manthiram K, Beberwyck BJ, Alivisatos AP (2014). Enhanced electrochemical methanation of carbon dioxide with a dispersible nanoscale copper catalyst. J. Am. Chem. Soc..

[24] Deen, W. *Analysis of Transport Phenomena, Topics in Chemical Engineering* (Oxford Univ. Press, 2012).

[25] Hsu S-H (2009). Bayesian framework for building kinetic models of catalytic systems. Ind. Eng. Chem. Res..

[26] Grinstead, C. M. & Snell, J. L. *Introduction to Probability* (American Mathematical Soc., 2012).

[27] Chandler, D. & Wu, D. *Introduction to Modern Statistical Mechanics* (Oxford Univ. Press, 1987).

[28] Zeng JS, Corbin N, Williams K, Manthiram K (2020). Kinetic analysis on the role of bicarbonate in carbon dioxide electroreduction at immobilized cobalt phthalocyanine. ACS Catal..

[29] Ringe S (2020). Double layer charging driven carbon dioxide adsorption limits the rate of electrochemical carbon dioxide reduction on Gold. Nat. Commun..

[30] Lim RJ (2014). A review on the electrochemical reduction of CO2 in fuel cells, metal electrodes and molecular catalysts. Catal. Today.

[31] Efron, B. & Tibshirani, R. J. *An Introduction to the Bootstrap* (CRC Press, 1994).

[32] Bockris JO, Nagy Z (1973). Symmetry factor and transfer coefficient. A source of confusion in electrode kinetics. J. Chem. Ed..

[33] Chandler, D. Electron transfer in water and other polar environments, how it happens. In *Classical and Quantum Dynamics in Condensed Phase Simulations*, 25–49 (1998).

[34] Guidelli R (2014). Defining the transfer coefficient in electrochemistry: an assessment (IUPAC Technical Report). Pure Appl. Chem..

[35] Limaye AM, Willard AP (2019). Modeling interfacial electron transfer in the double layer: the interplay between electrode coupling and electrostatic driving. J. Phys. Chem. C.

[36] Guidelli, R. & Schmickler, W. *Modern Aspects of Electrochemistry*, 303–371 (Springer, 2005).

[37] Adanuvor PK, White RE (1988). Analysis of electrokinetic data by parameter estimation and model discrimination technqiues. J. Electrochem. Soc..

[38] Rohatgi, A. *WebPlotDigitizer* https://automeris.io/WebPlotDigitizer (2017).

[39] Virtanen P (2020). SciPy 1.0: fundamental algorithms for scientific computing in Python. Nat. Methods.

[40] Salvatier J, Wiecki TV, Fonnesbeck C (2016). Probabilistic programming in Python using PyMC3. PeerJ Comput. Sci..

