# Supplementary material for: Bayesian data analysis reveals no preference for cardinal Tafel slopes in CO2 reduction electrocatalysis
Source: Nat Commun. 2021 Jan 29;12:703. doi: 10.1038/s41467-021-20924-y (PMC7846806; doi:10.1038/s41467-021-20924-y)
Supplement: Supplementary file 1 — Supplementary Information [file 41467_2021_20924_MOESM1_ESM.pdf]

Supporting Information: Bayesian data analysis reveals no preference for cardinal Tafel slopes in CO_2 reduction electrocatalysis

Aditya M. Limaye,† Joy S. Zeng,† Adam P. Willard,*,‡ and Karthish Manthiram*,†

†*Department of Chemical Engineering, MIT, Cambridge, MA*

‡*Department of Chemistry, MIT, Cambridge, MA*

E-mail: awillard@mit.edu; karthish@mit.edu

Supplementary Discussion

Literature Analysis

Dataset Organization

In this study, we manually digitized Tafel data included in several different papers from the electrochemical CO_2 reduction literature. Often, a single paper may report multiple sets of Tafel data, possibly with many traces in the same figure. Each distinct paper that reports (possibly multiple) Tafel data(sets) receives a unique three-digit identifier, which will be referred to as `PaperID`. Note that `PaperID`s are *not* guaranteed to be consecutive, and some numbers are skipped. Within each paper, each dataset receives a separate three-digit

identifier (unique within the same PaperID), which will be referred to as SetID. A single Tafel dataset in our study, then, is uniquely identified by the tuple (PaperID, SetID).

Our entire zipped dataset is organized into a hierarchical directory structure, with each directory named according to the PaperID identifier. Each individual paper directory contains several files. First, we include files with names of the form figure*.png, which are raw Portable Network Graphics (PNG) images of the paper figures, screenshotted manually from PDF copies of the papers. A few paper directories have multiple figure screenshots, and they are named according to figure1.png, figure2.png, etc. Directories also include raw data files with names of the form dat_<SetID>.txt, which contain raw x, y data taken from manually digitizing the figure. Associated with each raw data file is a separate metadata file with a name of the form metadata_<SetID>.txt. The metadata files contain key-value pairs stored in the YAML Ain't Markup Language (YAML) format, and contain several pieces of information required for processing the raw x, y data. YAML parsers are available in several programming languages, and the official YAML standard is documented at https://yaml.org/.

Metadata Tags

Tags in a metadata file are associated with YAML keys, and their associated values have several types. The types of the values, as well as the information they carry, are documented in Table 1. Note that for CO_2 reduction catalysis, more negative applied electrochemical potentials correspond to more positive overpotentials. Studies in the literature report either the applied electrochemical potential or the overpotential on their voltage axis. The v_reversed tag contains the information required to properly orient the voltage data in the direction of increasing overpotential for each dataset.

Supplementary Table 1: Table describing the types and meanings of the key-value pairs in the metadata files associated with each Tafel dataset.

Tag Name	Value Type	Value Meaning
`xdat`	Enum[‘`current`’, ‘`voltage`’]	Indicates which data is reported on the x axis.
`xunit`	`String`	String describing units of measurement for the x axis data, for example `mV` or `mA/cm2`.
`xlog`	`Bool`	Indicates whether the x axis data is reported on a logarithmic scale.
`ydat`	Enum[‘`current`’, ‘`voltage`’]	Indicates which data is reported on the y axis.
`yunit`	`String`	String describing units of measurement for the y axis data, for example `mV` or `mA/cm2`.
`ylog`	`Bool`	Indicates whether the y axis data is reported on a logarithmic scale.
`rval`	`Float`	Reported Tafel slope value.
`rerr`	Union[`Float`, `None`]	Error in reported Tafel slope value, if reported. If not reported, then `None`.
`runit`	`String`	String describing units of measurement for the x axis data, for example `mV/dec`.
`v_reversed`	`Bool`	If `false`, then the voltage data increases in value with increasing direction of the axis. If `true`, then the voltage data decreases in value with decreasing direction of the axis.
`i_reversed`	`Bool`	If `false`, then the current data increases in value with increasing direction of the axis. If `true`, then the current data decreases in value with decreasing direction of the axis.
`cat_tags`	List[`String`]	A list of tags describing the catalyst material. For example, a CuO catalyst would get the tags [‘`Cu`’, ‘`O`’].

Analyzed Papers

Table 2 documents the literature sources of all Tafel datasets analyzed in this study.

Supplementary Table 2: Provenance of all Tafel datasets analyzed in this study. The `PaperID` defines the unique identifier assigned to the paper in the zipped dataset.

PaperID	Data Location	Document Object Identifier (DOI)
000	Figure 5	10.1021/ja5065284[1]
002	Figure 4	10.1021/ja309317u[2]
004	Figure 4	10.1246/bcsj.68.1889[3]
005	Figure 4	10.1002/anie.201604654[4]
009	Figure 5B	10.1021/acsnano.5b01079[5]
010	Figure 4B	10.1002/anie.201713003[6]
011	Figure 1C	10.1021/jacs.7b09074[7]
012	SI Figure 8	10.1002/anie.201900499[8]
014	Figure 10	10.1002/smll.201701809[9]
015	Figure 6D	10.1088/1361-6528/aa8f6f[10]
016	Figure 7A	10.1021/jacs.5b02975[11]
017	SI Figure 6	10.1002/smll.201602158[12]
021	Figure 5C	10.1002/celc.201700517[13]
022	Figure 3A, 3C	10.1002/cssc.201600202[14]
023	Figure 4A	10.1021/ja501923g[15]
024	Figure 4B	10.1021/acsenergylett.8b00472[16]
025	SI Figure 12	10.1021/ja4113885[17]
026	Figure 2D	10.1016/j.elecom.2016.05.003[18]
027	Figure 3A, 3C	10.1021/ja2108799[19]
028	Figure 7A	10.1021/acs.jpcc.7b01586[20]
029	SI Figure 13	10.1021/acscatal.7b00707[21]
031	Figure 7	10.1016/j.electacta.2016.03.182[22]

032	Figure 8	10.1016/j.jcou.2017.05.024[23]
033	Figure 2D	10.1002/cssc.201902859[24]
035	Figure 4	10.1021/acsami.8b03461[25]
036	SI Figure 8	10.1021/acsami.7b10421[26]
039	Figure 3A	10.1002/asia.201800946[27]
040	Figure 7	10.1002/cssc.201802409[28]
042	SI Figure 4	10.1021/acsaem.8b00356[29]
043	SI Fiigure 14	10.1021/acs.jpcc.8b06234[30]
045	SI Figure 13	10.1021/acsaem.8b02048[31]
048	Figure 6C	10.1002/celc.201801132[32]
049	Figure 4C	10.1002/adma.201706194[33]
050	Figure 3	10.1038/ncomms4242[34]
051	Figure 4	10.1021/acscatal.5b01235[35]
052	Figure 4E	10.1002/aenm.201701456[36]
053	Figure 3F	10.1016/j.chempr.2017.08.002[37]
054	Figure 4C	10.1002/anie.201608279[38]
055	Figure 7A	10.1016/j.apcatb.2018.01.001[39]
056	Figure 4	10.1021/ja3010978[40]
057	Figure 2C	10.1016/j.apcatb.2018.09.025[41]
058	Figure 5D	10.1021/jacs.6b10435[42]
059	Figure 6	10.1021/jacs.6b12217[43]
060	Figure 2F	10.1002/ange.201805696[44]
061	Figure 6	10.20964/2017.03.72[45]
062	Figure 3B	10.1002/cssc.201702229[46]
063	Figure 3D	10.1002/aenm.201801536[47]
064	Figure 4A	10.1016/j.jcis.2018.09.036[48]
065	Figure 7	10.1016/j.nanoen.2018.03.023[49]

066	Figure 3D	10.1021/jacs.7b12506[50]
067	Figure 2D	10.1021/acscatal.5b00922[51]
068	Figure 3F	10.1021/acsnano.7b03029[52]
069	Figure 4D	10.1021/acscatal.7b03449[53]
070	Figure 8	10.1016/j.apsusc.2016.10.017[54]
071	Figure 3	10.1002/anie.201802055[55]
072	Figure 6A	10.1016/j.electacta.2018.04.047[56]
074	Figure 2G	10.1021/jacs.5b08212[57]
076	Figure 3A	10.1002/anie.201809255[58]
077	Figure 4A	10.1002/anie.201711255[59]
078	Figure 3G	10.1016/j.nanoen.2018.09.053[60]
080	Figure 2H	10.1002/anie.201800367[61]
081	Figure 7	10.1016/j.cej.2016.02.084[62]
083	Figure 2B	10.1016/j.apcatb.2017.06.032[63]
084	Figure 2E	10.1016/j.jcat.2018.05.005[64]
085	Figure 3E	10.1002/anie.201803873[65]
086	Figure 3C	10.1002/anie.201806043[66]
087	Figure 4D	10.1016/j.electacta.2018.09.080[67]
088	Figure 5	10.1023/B:JACH.0000003866.85015.b6[68]
089	Figure 6	10.1021/jp9822945[69]
090	Figure 5C	10.1002/smll.201704049[70]
091	Figure 5D	10.1016/j.nanoen.2016.11.004[71]
092	Figure 5B	10.1002/anie.201701104[72]
093	Figure 3A	10.1021/jacs.6b12103[73]
094	Figure 4A	10.1002/anie.201903613[74]
095	SI Figure 8C	10.1002/anie.201900499[8]
096	Figure 2B	10.1002/adfm.201800499[75]

097	SI Figure 6	10.1016/j.cattod.2015.05.017[76]
098	Figure 4C	10.1021/acsami.7b16164[77]
099	Figure 3	10.1002/smll.201703314[78]
100	Figure 4A	10.1016/j.apcatb.2018.08.075[79]
101	Figure 4D	10.1073/pnas.1711493114[80]
102	Figure 5B	10.1002/cssc.201800925[81]
103	Figure 1C	10.1021/acscatal.5b02424[82]
104	Figure 3F	10.1021/acsenergylett.8b00519[83]
105	Figure 4B	10.1002/anie.201612194[84]
106	Figure 3C	10.1002/anie.201703720[85]
108	SI Figure 4	10.1021/acs.nanolett.5b04123[86]
109	Figure 3F	10.1002/anie.201901575[87]
110	Figure 3D	10.1016/j.electacta.2018.12.116[88]
111	Figure 5A	10.1021/acscatal.8b01022[89]
112	Figure 3	10.1002/cssc.201701673[90]
113	Figure 2D	10.1002/aenm.201900276[91]
114	Fgiure 2D	10.1021/acsenergylett.8b01286[92]
115	Figure 2D	10.1002/anie.201712221[93]
116	Figure 2D	10.1002/anie.201807571[94]
117	Figure 3A	10.1021/acsenergylett.7b01096[95]
118	Figure 5	10.1021/acsaem.8b01692[96]
119	Figure 6	10.1039/C5CP03559G[97]
120	SI Figure 6	10.1126/science.aaw7515[98]
121	Figure 10	10.1002/celc.201801036[99]
122	Figure 5A	10.1002/adma.201705872[100]
123	Figure 4C	10.1021/acscatal.8b04852[101]
124	Figure 2F	10.1016/j.jcou.2019.05.026[102]

125	Figure 3D	10.1002/ange.201810538[103]
126	Figure 3C	10.1016/j.joule.2018.11.008[104]
127	Figure 1D	10.1002/aenm.201700759[105]
128	Figure 5B	10.1002/celc.201700935[106]
129	Figure 4B	10.1016/j.apcatb.2019.03.047[107]
130	SI Figure 5B	10.1002/anie.201911995[108]
131	SI Figure 12	10.1002/aenm.201702524[109]
132	Figure 4C	10.1002/ange.201805256[110]
133	SI Figure 10	10.1002/aenm.201601103[111]
134	Figure 2A	10.1038/s41467-018-07970-9[112]
135	Figure 2B	10.1016/j.nanoen.2019.05.003[113]
136	Figure 4	10.1039/C6TA04325A[114]
137	Figure 2D	10.1002/celc.201800806[115]
138	Figure 3B	10.1002/anie.201805871[116]
139	Figure 4	10.1002/chem.201603359[117]
140	Figure 3D	10.1002/aenm.201903068[118]
141	Figure 3	10.1002/cssc.201501637[119]
142	Figure 3F	10.1002/anie.201907399[120]
143	Figure 3E	10.1016/j.electacta.2018.08.002[121]
144	Figure 4A	10.1021/jp509967m[122]
145	SI Figure 8	10.1021/acs.est.5b00066[123]
146	SI Figure 10	10.1021/acscatal.6b00543[124]
147	SI Figure 39	10.1016/j.joule.2019.05.010[125]
148	Figure 3B	10.1016/j.jcou.2019.02.007[126]
149	Figure 4D	10.1002/cssc.201903117[127]
150	Figure 3C	10.1002/chem.201803615[128]
151	Figure 5D	10.1002/celc.201800104[129]

152	Figure 4A	10.1039/C9EE00018F[130]
153	SI Figure 10	10.1016/j.joule.2018.10.015[131]
154	Figure 5	10.1002/celc.201900725[132]
155	Figure 4B	10.1038/ncomms14503[133]
156	Figure 2D	10.1016/j.elecom.2018.10.014[134]
157	Figure 2C	10.1002/aenm.201803151[135]
158	Figure 4E	10.1016/j.elecom.2019.03.017[136]
159	Figure 4B, 4C	10.1002/cssc.201802725[137]
160	Figure 12	10.1016/j.matchemphys.2017.02.016[138]
161	Figure 4	10.1002/adfm.201802339[139]
162	Figure 4D	10.1016/j.nanoen.2016.06.043[140]
163	Figure 4C	10.1038/ncomms12697[141]
164	Figure 4F	10.1021/acssuschemeng.9b03502[142]
165	Figure 5B	10.1039/C8TA03328E[143]
166	Figure 6C	10.1039/C7TA03005C[144]
167	Figure 3A	10.1002/anie.201908735[145]
168	Figure 6D	10.1021/acsaem.9b02324[146]
169	Figure 2C	10.1021/acs.jpcb.9b09730[147]
170	Figure 3A	10.1073/pnas.1602984113[148]
171	Figure 2	10.1021/acscatal.8b02181[149]

Data Licensing

Code that supports the findings of this study is available under the MIT License in Zenodo (`http://doi.org/10.5281/zenodo.3995021`). Data that supports the findings of this study is available under CC BY 4.0 (`https://creativecommons.org/licenses/by/4.0/`) in Zenodo (`http://doi.org/10.5281/zenodo.3995021`), with the exception of the excerpted figures from other articles as described in the Supporting Information. The excerpted fig-

ures are reused under agreement between MIT and the publishers of the articles (`https://libraries.mit.edu/scholarly/publishing/using-published-figures/`), where the copyright is owned by the publishers.

Residuals vs. Literature-Reported Values

Residual analysis can help sniff out systematic or correlated errors in the results presented in Fig. 3A of the main text. We can define residuals between the literature-reported values and the MAP values in several different ways. Here, we will consider two definitions of the residuals, normalized either to the literature-reported Tafel slopes,

$$\text{Residual Normalized to Reported} = \frac{T_{\text{reported}} - T_{\text{MAP}}}{T_{\text{reported}}}, \tag{1}$$

or to the MAP Tafel slopes,

$$\text{Residual Normalized to MAP} = \frac{T_{\text{reported}} - T_{\text{MAP}}}{T_{\text{MAP}}}. \tag{2}$$

Figure 1 depicts plots of the residuals versus the literature-reported and MAP Tafel slopes (A and C, respectively), as well as kernel density estimates of the distribution over the residuals normalized to the literature-reported and MAP Tafel slopes (B and D, respectively). To the eye, the residuals appear roughly unbiased around zero, and there appear to be no spurious correlations between the residuals.

Correlation Plot over Full Range

Figure 2 depicts a correlation plot of the MAP Tafel slope versus the literature-reported Tafel slope, including all datasets considered in this study.

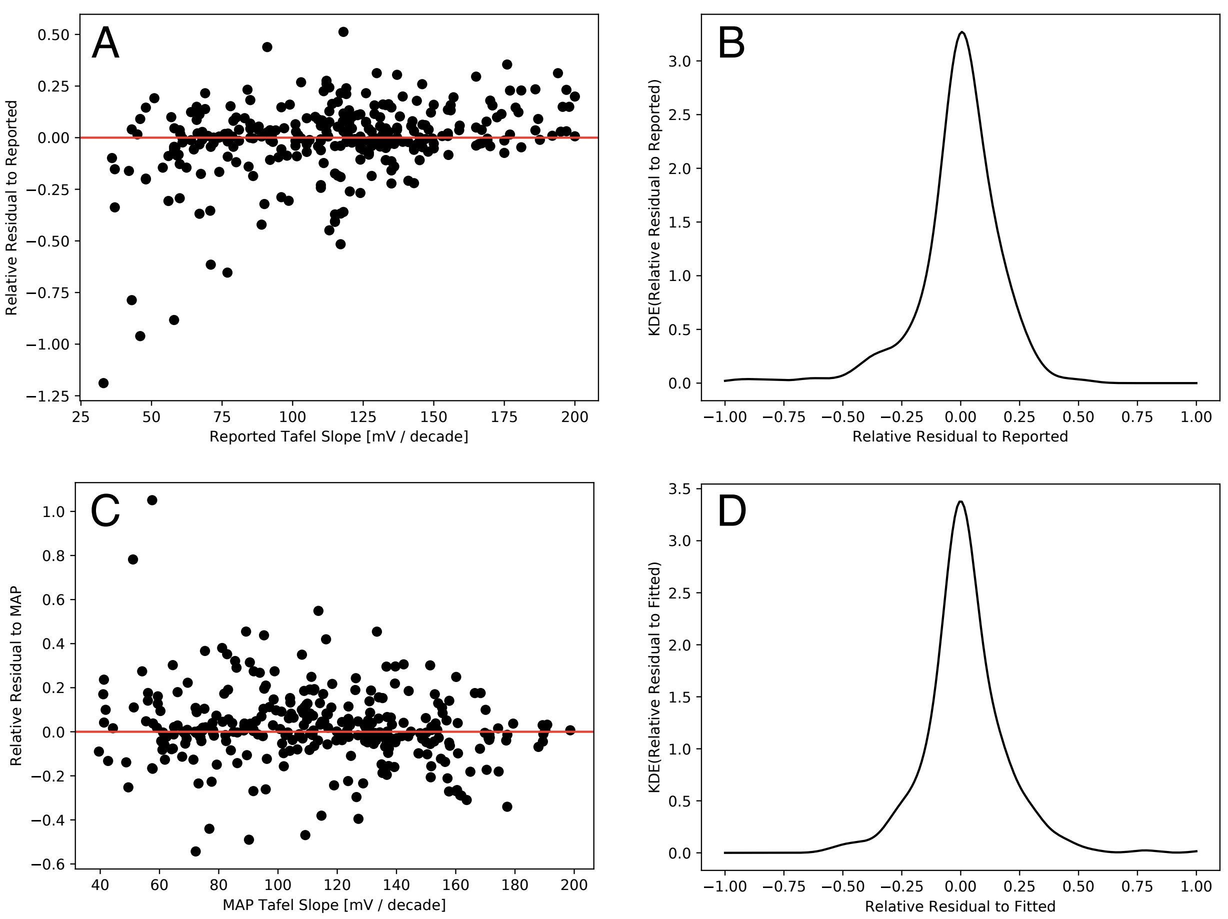

Supplementary Figure 1: Residual Analysis. (A) Plot of the relative residual to the literature-reported Tafel slope, defined in Eq. (1), versus the literature-reported Tafel slope, including only reported Tafel slopes less than 200 mV/decade. (B) Kernel density estimate of the distribution over the relative residual to the literature-reported Tafel slope. (C) Plot of the relative residual to the MAP Tafel slope, defined in Eq. (1), versus the MAP Tafel slope, including only reported Tafel slopes less than 200 mV/decade. (D) Kernel density estimate of the distribution over the relative residual to the MAP Tafel slope.

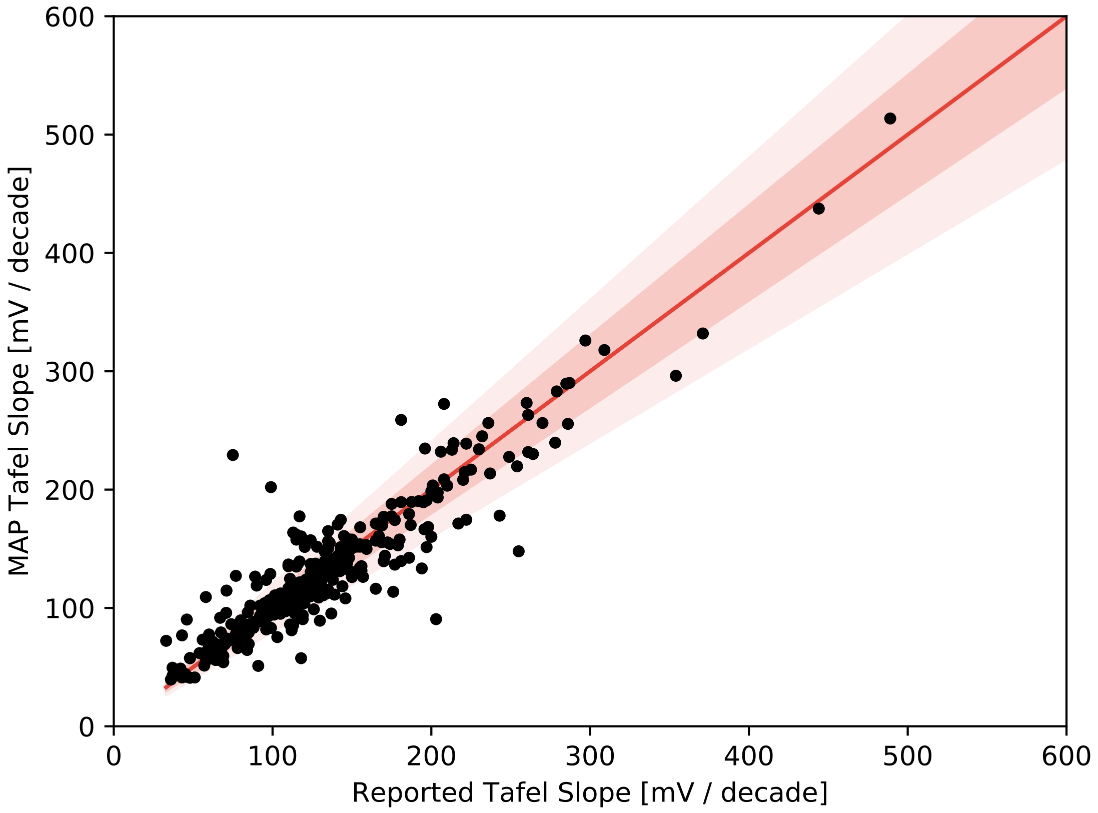

Supplementary Figure 2: Correlation plot of reported Tafel slopes from the literature against Tafel slopes fitted by our algorithm on identical data. The solid red line represents perfect agreement, while the red filled intervals are lines representing 10% and 20% relative error.

PDF Plot Using all Posterior Samples

For every single dataset considered in the study, we draw $N = 4 \times 10^4$ samples from the posterior distribution over the Tafel slope. In Fig. 3C in the main text, we depict a kernel density estimate of the distribution over the MAP Tafel slope from each of these posterior distributions. We choose to display this data because the MAP Tafel slope is a straightforward point estimate of the Tafel slope given a posterior distribution over the parameter, and hence is likely the quantity one would quote if asked what *the* Tafel slope of a catalyst is. However, the averaging operation involved in computing the MAP Tafel slope can collapse broad features in the posterior distribution down to a single value; this is especially true in the case of bimodal posterior distributions arising from insufficient datasets. Figure 3 depicts a kernel density estimate of the distribution over the Tafel slope using all samples collected from the posterior distribution for each dataset. The essential conclusions reported in the main text are upheld when examining the data in Fig. 3. A very small preference for Tafel slopes around 45 mV/decade emerges in this analysis. However, given the small amount of total distributional mass under this peak and the high degree of sampling variability (as evinced by the bootstrap standard deviations), we do not believe it should be interpreted strongly.

Catalyst Breakout Results

As described in the main text, we split out our results on the distributions over the Tafel slope by catalyst material identity in order to confirm that our conclusion of a lack of Tafel cardinality in CO_2 reduction catalysis is not an artifact of pooling together data from several catalyst materials, each of which individually exhibit cardinality. Figures 4–9 depict these results for catalysts containing Cu, Ag, Au, Sn, and Bi. Each figure has three panels: the left-most panel plots a kernel density estimate of the distribution over Tafel slopes using all samples from the posterior distribution, akin to Fig. 3. The center panel plots a kernel density estimate of the distribution over the MAP Tafel slope from each dataset, akin to main

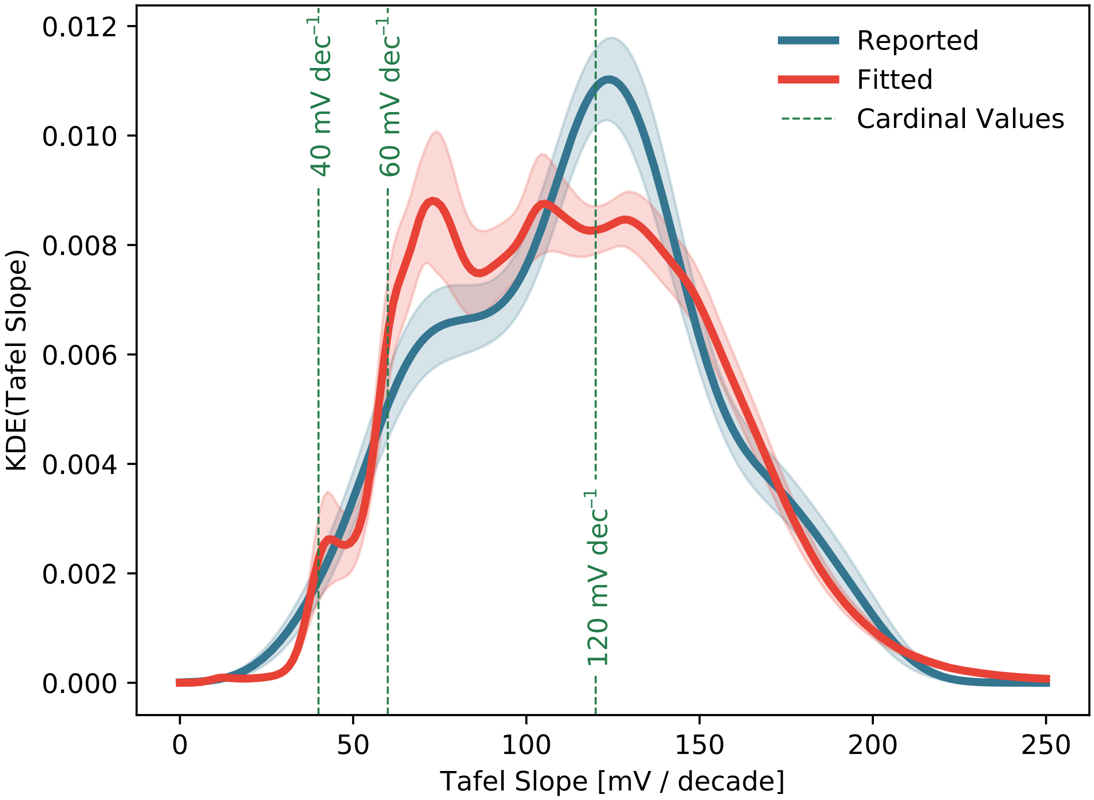

Supplementary Figure 3: Kernel density estimates (KDE) of the empirical probability distribution function of Tafel slopes reported in literature data (blue) and those refitted by our algorithm (red). Error intervals correspond to one standard deviation of bootstrapped resamples. Green dashed lines correspond to cardinal values of the Tafel slope.

text Fig. 3C. The right-most panel plots a CDF of the distributional breadth B_i for each dataset considered. The distributional breadth is parametrized by the threshold parameter t, and is defined as,

$$B_i(t) = \mathrm{CDF}_i^{-1}(1-t) - \mathrm{CDF}_i^{-1}(t), \tag{3}$$

where $\mathrm{CDF}_i(m_\mathrm{T})$ represents the CDF of the Tafel slope for the i'th dataset. Intuitively, a high distributional breadth implies that the experimental data measured in the dataset does not provide information to pin down the value of the Tafel slope with high confidence. Correspondingly, if the CDF of the distributional breadth increases quickly, then most of the datasets in the catalyst material subset predict tight distributions over the Tafel slope. Conversely, if the CDF of the distributional breadth climbs slowly, then several datasets in the catalyst material subset do not determine a Tafel slope value with high confidence.

For some catalysts, the KDE constructed from all Tafel slope samples (left panel) and the KDE constructed from the MAP Tafel slope samples (center panel) show different behavior. The former plot looks more visually noisy than the latter plot; this is to be expected, since taking the mean of the posterior distribution over the parameters is a "smoothing" operation. The two ways of visualizing the data convey slightly distinct information, since the KDE constructed from all samples preserves the uncertainty information retained in a single fit, while the KDE constructed from the MAP Tafel slopes is the most direct comparison to the distribution of literature values (which do not carry an associated uncertainty, in most cases). In certain cases (Ag, Sn), the KDE comprising all samples appears to have more defined peaks than the KDE comprising MAP samples. First, we note that in these cases, the bootstrap standard errors for these peaks are much greater than in other areas of the distribution, suggesting that this peaking behavior is controlled by a small number of samples, and hence more variable with respect to a change in the specific datasets re-analyzed in this study. Second, peaking behavior in the KDE comprising all samples that does not appear in the KDE comprising MAP samples is a sign of some underlying data insufficiency issues highlighted by Fig. 2 in the main text; these peaks can easily disappear

or be shifted upon measuring additional data. To declare confidently that a certain dataset espouses a cardinal Tafel slope preference, we contend that *both* ways of visualizing the distribution of Tafel slopes for a certain catalyst should show peaking around a cardinal value. This standard, while stringent, enforces that the available experimental data confidently determines a cardinal value of the Tafel slope, free of latent data insufficiency issues. While we observe this for the Bi breakout results in Fig. 9, the remainder of the catalyst breakout datasets do not meet this standard. Hence, though we do not foreclose the possibility that additional future data collection on these catalysts may reveal a cardinal Tafel slope preference, we argue that broadly, when considering the data extant in the literature, the typical CO_2 reduction catalyst does not exhibit a strong preference for cardinal values of the Tafel slope.

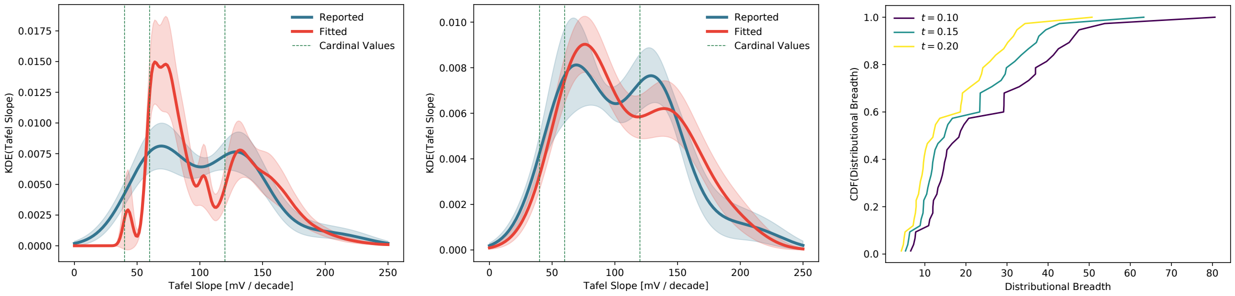

Supplementary Figure 4: Tafel Slope statistics for catalysts containing Ag ($N_{\text{datasets}} = 38$). (Left) Kernel density estimates (KDE) of the empirical probability distribution function of Tafel slopes reported in literature data (blue) and those refitted by our algorithm (red). Error intervals correspond to one standard deviation of bootstrapped resamples. Green dashed lines correspond to cardinal values of the Tafel slope. (Center) Same as (Left), but KDEs are computed using only the MAP Tafel Slope values for each dataset. (Right) CDF of the distributional breadth, as defined by Eq. (3).

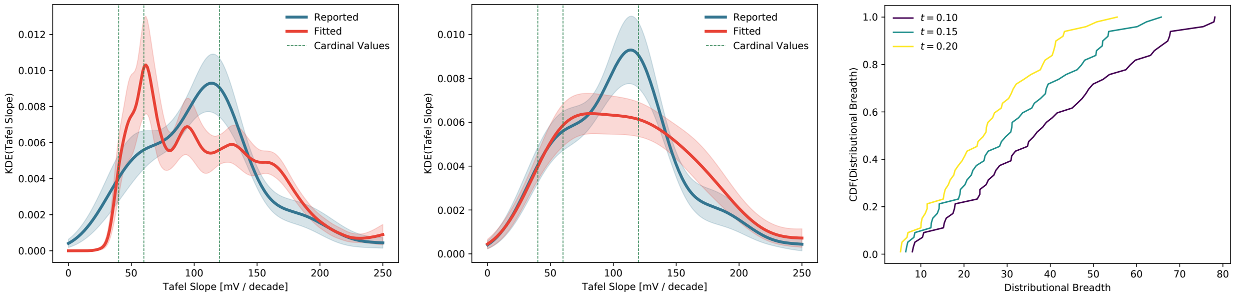

Supplementary Figure 5: Tafel Slope statistics for catalysts containing Au ($N_{\text{datasets}} = 50$). (Left) Kernel density estimates (KDE) of the empirical probability distribution function of Tafel slopes reported in literature data (blue) and those refitted by our algorithm (red). Error intervals correspond to one standard deviation of bootstrapped resamples. Green dashed lines correspond to cardinal values of the Tafel slope. (Center) Same as (Left), but KDEs are computed using only the MAP Tafel Slope values for each dataset. (Right) CDF of the distributional breadth, as defined by Eq. (3).

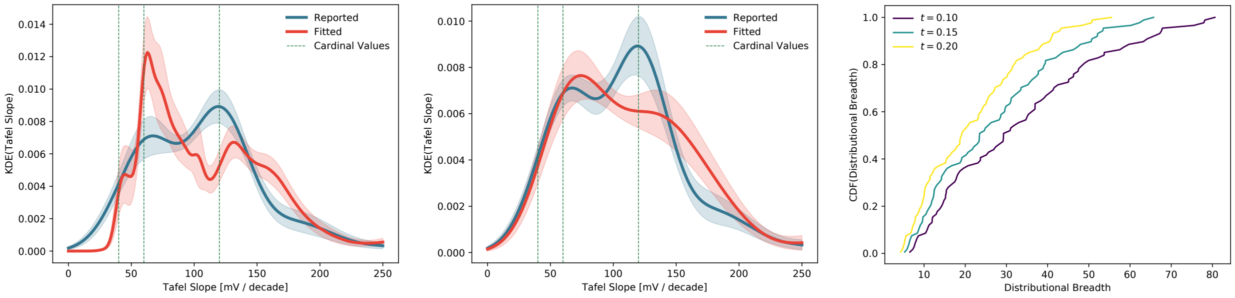

Supplementary Figure 6: Tafel Slope statistics for catalysts containing Ag or Au ($N_{\text{datasets}} = 88$). (Left) Kernel density estimates (KDE) of the empirical probability distribution function of Tafel slopes reported in literature data (blue) and those refitted by our algorithm (red). Error intervals correspond to one standard deviation of bootstrapped resamples. Green dashed lines correspond to cardinal values of the Tafel slope. (Center) Same as (Left), but KDEs are computed using only the MAP Tafel Slope values for each dataset. (Right) CDF of the distributional breadth, as defined by Eq. (3).

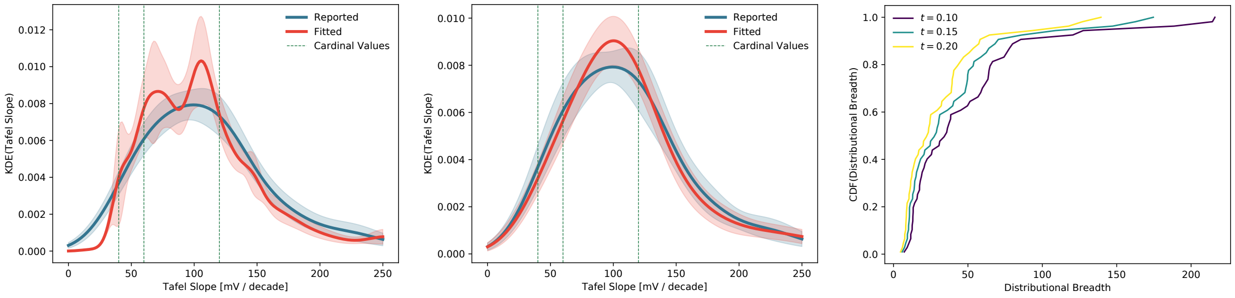

Supplementary Figure 7: Tafel Slope statistics for catalysts containing Cu ($N_{\text{datasets}} = 54$). (Left) Kernel density estimates (KDE) of the empirical probability distribution function of Tafel slopes reported in literature data (blue) and those refitted by our algorithm (red). Error intervals correspond to one standard deviation of bootstrapped resamples. Green dashed lines correspond to cardinal values of the Tafel slope. (Center) Same as (Left), but KDEs are computed using only the MAP Tafel Slope values for each dataset. (Right) CDF of the distributional breadth, as defined by Eq. (3).

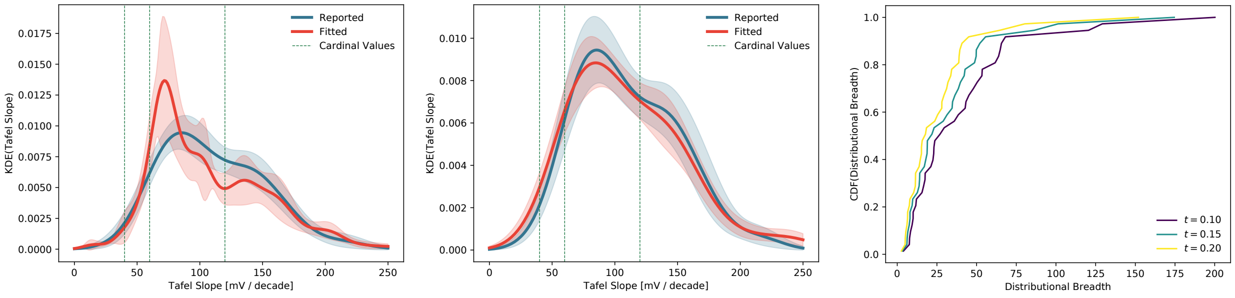

Supplementary Figure 8: Tafel Slope statistics for catalysts containing Sn ($N_{\text{datasets}} = 37$). (Left) Kernel density estimates (KDE) of the empirical probability distribution function of Tafel slopes reported in literature data (blue) and those refitted by our algorithm (red). Error intervals correspond to one standard deviation of bootstrapped resamples. Green dashed lines correspond to cardinal values of the Tafel slope. (Center) Same as (Left), but KDEs are computed using only the MAP Tafel Slope values for each dataset. (Right) CDF of the distributional breadth, as defined by Eq. (3).

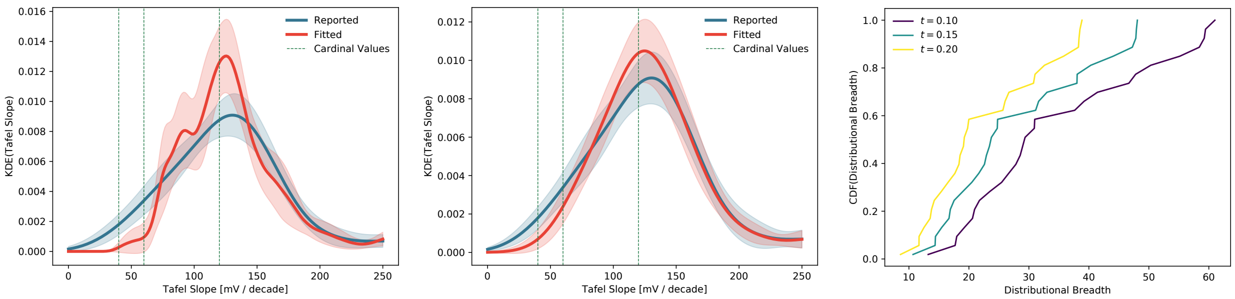

Supplementary Figure 9: Tafel Slope statistics for catalysts containing Bi ($N_{\text{datasets}} = 27$). (Left) Kernel density estimates (KDE) of the empirical probability distribution function of Tafel slopes reported in literature data (blue) and those refitted by our algorithm (red). Error intervals correspond to one standard deviation of bootstrapped resamples. Green dashed lines correspond to cardinal values of the Tafel slope. (Center) Same as (Left), but KDEs are computed using only the MAP Tafel Slope values for each dataset. (Right) CDF of the distributional breadth, as defined by Eq. (3).

Limiting Current Statistics

Figure 10 depicts kernel density estimates of the distribution of fitted limiting currents i_{lim} from a re-analysis of literature data. Roughly, the distribution appears to be in agreement with the value of the transport-limited current density for CO_2 reduction at an aqueous flooded electrode.[150]

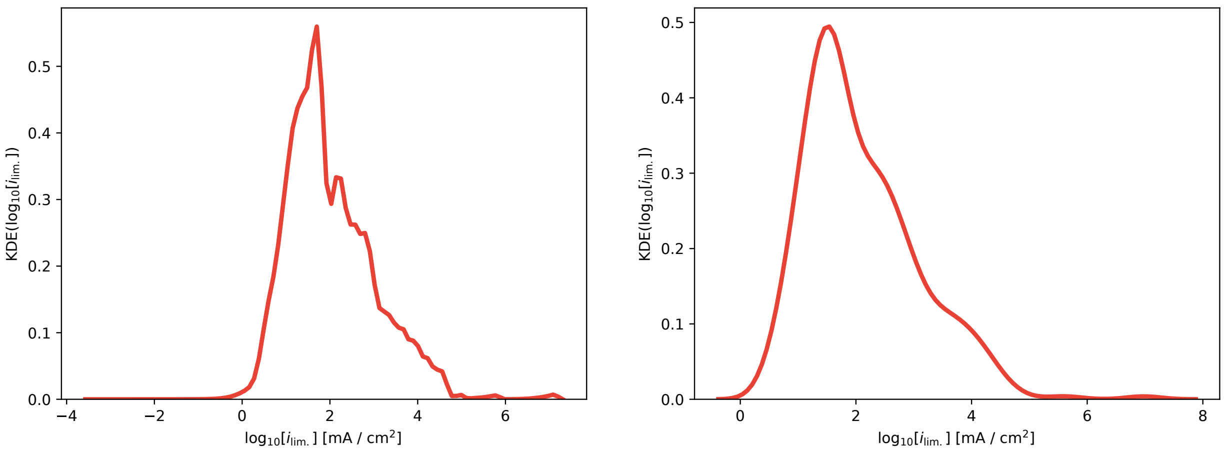

Supplementary Figure 10: Limiting current statistics for all datasets analyzed in the study. (Left) Kernel density estimates (KDE) of the empirical probability distribution of the limiting current, i_{lim} fitted when interpreting the data through the model in main text Eq. (2). (Right) Same as (Left), but KDEs are computed using only the MAP limiting current values for each dataset.

Bayesian Fitting

Mathematical Detail

As quoted in the main text, Bayes' rule reads,

$$p(\theta|\mathbf{y}) = \frac{p(\mathbf{y}|\theta) \times p(\theta)}{p(\mathbf{y})}. \tag{4}$$

In the context of this work, $\mathbf{y}$ represents the measured current data at a set of voltage points. We will use the subscript notation $\mathbf{y}_k$ to denote a single current data point, where the index $k = 1, \ldots, N_{\text{pts}}$. The parameters of a model for interpreting current-voltage data are denoted

by θ; in the context of this work, the relevant parameters for the limiting current model are $i_{\rm lim}$, the limiting current density, i_0, the exchange current density, and $m_{\rm T}^{-1}$, the inverse Tafel slope. We will denote the model's predictions at each voltage point by the subscript notation $\mathcal{M}_k(\theta)$.

To successfully apply Bayes' rule to glean $p(\theta|\mathbf{y})$, the posterior distribution over the model parameters given measured data, we need to identify mathematical forms for the prior distribution $p(\theta)$ and the likelihood function $p(\mathbf{y}|\theta)$. In all fits conducted in this study, we employ a uniform prior distribution (also known as an "uninformative" prior distribution) over a certain parameter range. Since the prior is uniform in the selected parameter range, as long as the range includes the values of the parameters for which $p(\theta|\mathbf{y})$ has high probability mass, the choice of prior is unimportant (see further on for a numerical confirmation of this fact). Given this fact, the choice for the range of our uniform prior is determined by first using a standard nonlinear least-squares optimization algorithm (TRF) to determine a point estimate of the optimal set of parameters, θ^*. Formally,

$$\theta^* = \arg\min_{\theta} \left\{ \sum_{k=1}^{N_{\rm pts}} \left[\mathbf{y}_k - \mathcal{M}_k(\theta)\right]^2 \right\}. \tag{5}$$

With the optimal parameters θ^* in hand, we select a uniform prior $p(\theta)$ that is supported in the range $[0, a \times \theta_i^*]$ for each parameter $i = 1, \ldots, N_{\rm params}$. We choose the *very* conservative value $a = 10$ to ensure that the prior distribution has support over a very broad range around the optimal parameters. In principle, this choice of a results in a wide parameter space, which may affect the computational efficiency of a posterior sampling algorithm. In practice, we find very little computational disadvantage for choosing $a = 10$ as compared to $a = 2$ when using the No-U-Turn Sampler implemented in PyMC3.

The likelihood function for the data given the parameters, $p(\mathbf{y}|\theta)$, is determined by assuming that the experimental measurement represents a ground truth measurement described

by the model, polluted by unavoidable experimental error,

$$\mathbf{y}_k = \mathcal{M}_k(\theta) + \epsilon_k. \tag{6}$$

We assume that errors at different data points are uncorrelated, and further assume that the error ϵ_i at any single data point is drawn from a Gaussian distribution with zero mean and variance σ^2,

$$p(\epsilon_k) = \frac{1}{\sqrt{2\pi}\sigma} \exp\left[-\frac{\epsilon_k^2}{2\sigma^2}\right]. \tag{7}$$

Because the errors at each point are uncorrelated, the likelihood now factorizes over all the data points,

$$p(\mathbf{y}|\theta) = \prod_{k=1}^{N_{\text{pts}}} \frac{1}{\sqrt{2\pi}\sigma} \exp\left[-\frac{(\mathbf{y}_k - \mathcal{M}_k(\theta))^2}{2\sigma^2}\right]. \tag{8}$$

With a likelihood function $p(\mathbf{y}|\theta)$ and a prior distribution $p(\theta)$ in hand, we can plug this information into a Monte Carlo sampler of our choice to draw samples from the posterior distribution $p(\theta|\mathbf{y})$. Equation (8) also makes apparent how one can generalize the Bayesian posterior sampling approach to more general models $\mathcal{M}(\theta)$. After we write down a suitable model, we simply evaluate the model predictions with different parameters whenever we need to compute the likelihood function for a given set of parameters during the sampling procedure.

Gaussian Error Estimates from Nonlinear Optimization

The Bayesian posterior sampling approach advanced in this work provides a way to glean distributional uncertainty information about the estimated values of model parameters given observed data. A similar set of information can also be obtained by analyzing the Hessian matrix determined by a nonlinear optimization algorithm seeking an optimal point estimate

of the model parameters. Specifically, if the optimizer seeks to minimize a loss function,

$$\mathcal{L}(\theta) = \frac{1}{2\sigma^2} \sum_{k=1}^{N_{\text{pts}}} \left[\mathbf{y}_k - \mathcal{M}_k(\theta)\right]^2, \tag{9}$$

then it often also produces an estimate of the Hessian,

$$\mathbf{H}_{ij} = \left. \frac{\partial \mathcal{L}}{\partial \theta_i \partial \theta_j} \right|_{\theta^*}, \tag{10}$$

evaluated at the optimal value of the parameters θ^*. If we assume that the experimental data is generated by the random process described by Eq. (6), and further assume that the errors at different data points are uncorrelated and drawn from Gaussian distributions with mean zero and variance σ^2, then we can form a Gaussian approximation to the posterior distribution around θ^*,

$$p(\theta|\mathbf{y}) \approx \frac{1}{(2\pi)^{d/2} (\det \mathbf{H})^{1/2}} \exp\left[-\frac{1}{2} (\theta - \theta^*)^{\mathrm{T}} \mathbf{H} (\theta - \theta^*)\right], \tag{11}$$

where d is the number of parameters being estimated, and $\mathbf{H}$ is guaranteed to be positive definite by virtue of being evaluated at the optimal point θ^*.

We stress that the expression provided by Eq. (11) is an *approximation* to the true posterior distribution; due to its Gaussian form, this expression can never accurately represent bimodality in the posterior distribution. In this sense, the Bayesian sampling approach is superior, although it comes at significant additional computational expense as d increases. In this work, $d = 3$, and this additional expense is essentially negligible given the computational power available on a typical laptop or desktop computer. Hence, we suggest that posterior distributions over the Tafel slope fitted using the model in Eq. (2) of the main text should always be computed using the Bayesian posterior sampling algorithm described in the previous section. We have simply included mention of the Gaussian approximation for the sake of completeness, and to guide possible future work that attempts to fit models

with significantly more parameters.

Sensitivity to Error Distribution Width

One important parameter of the Bayesian fitting approach is the width of the normal distribution governing the probability of deviations from the model, which arises when evaluating the quantity $p(\mathbf{y}|\theta)$ in Bayes' rule. Figure 11 studies the sensitivity of the posterior distribution over the Tafel slope to the parameter σ, the standard deviation of the normal distribution governing the statistics of the model error. As expected, lowering the value of

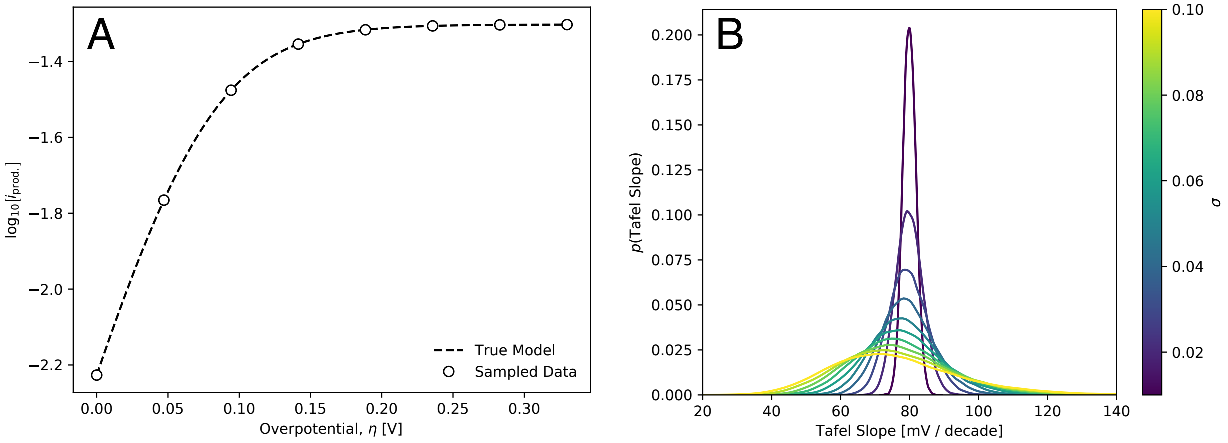

Supplementary Figure 11: Sensitivity to the width of the error distribution at each point for a simple fit to synthetic data. (A) Synthetic data sampled from a model with an underlying Tafel slope of 80 mV/decade. (B) Several traces of the posterior distribution computed using different values of σ, the standard deviation of the normal error distribution at each point.

σ causes the algorithm to become more confident in its estimate. At $\sigma = 0.01$ logarithmic units, the model essentially nails the true Tafel slope of 80 mV/decade. For larger values of σ, a clear distributional drift to lower values of the Tafel slope is observed. This occurs because most of the data in Fig. 11A lies in the plateau region, and the model faces less penalty for down-weighting these points as the value of σ is increasing. Hence, the model drifts to larger slopes on the Fig. 11A plot, which corresponds to lower values of the Tafel slope. Note that the distributional widening is significantly greater than the drift in the mean, suggesting that we should not put much stock into the mean drift. The upshot: for high values of the

σ parameter, this particular set of data does not contain enough information to pin down the value of the Tafel slope accurately.

Sensitivity to Prior Distribution

As explained in the main text, our Bayesian approach requires specification of a prior distribution $p(\theta)$ over the parameters. Since we know very little about the true distribution of the Tafel slope at the outset, we choose an uninformative uniform box prior over the interval $[0, a \times \theta_i^*]$ for each parameter θ_i, where θ_i^* is the optimal value of the parameter gleaned from the TRF algorithm described in the Methods section. Here, we conduct a sensitivity analysis on the a parameter. Given that we are using an uninformative prior, we should expect that the prior width should not influence the posterior distribution as long as the data expresses some opinion about the ideal value of the parameters. Figure 12 depicts the results of the sensitivity analysis on a set of synthetic data. Indeed, as expected, the posterior distribu-

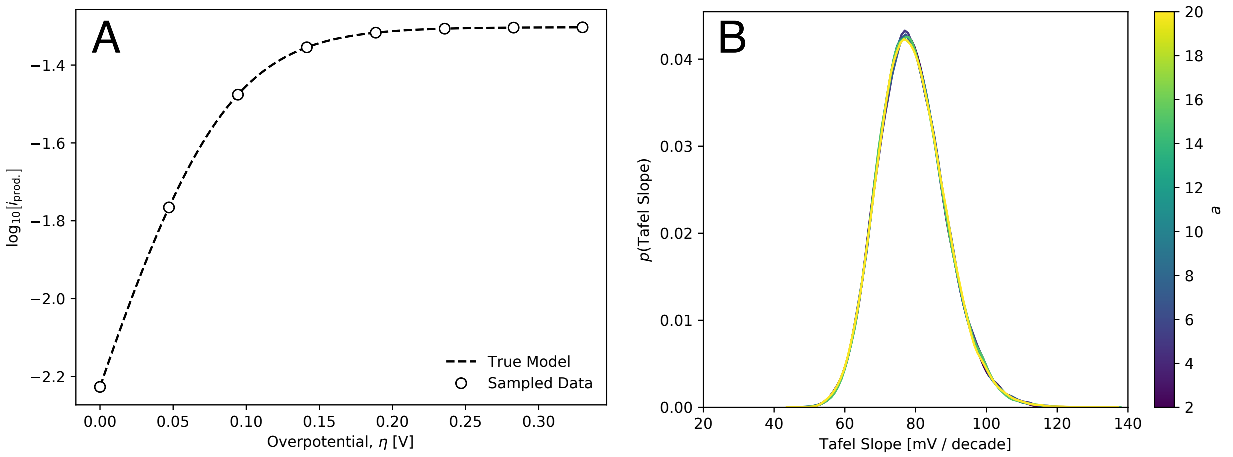

Supplementary Figure 12: Sensitivity to the width of the uniform prior distribution bounds. (A) Synthetic data sampled from a model with an underlying Tafel slope of 80 mV/decade. (B) Several traces of the posterior distribution computed using different values of a, a parameter influencing the width of the prior distribution fed to the Bayesian posterior sampling algorithm. All posterior distributions are computed with $\sigma = 0.05$.

tions are insensitive to the choice of prior. Note that in all fits considered in the main text, we use a value of $a = 10$, as mentioned in the Methods section.

Derivations

Cardinal Tafel Slopes Equation

We will work with a generic reaction scheme, assuming that we begin with a starting species $A^{(n+q)+}$ which undergoes n electron transfers prior to the rate-determining step, and then q electron transfers at the RDS. In practice n will be an integer, and q will be either zero or one. Here we assume the reaction taking place is reductive; however, the derivation is entirely analogous for an equivalent oxidation reaction. Schematically, the reactions read,

$$\begin{aligned} \mathrm{A}^{(n+q)+} + \mathrm{e}^- &\rightleftharpoons \mathrm{A}^{(n+q-1)+} \\ &\vdots \\ \mathrm{A}^{(q+1)+} + \mathrm{e}^- &\rightleftharpoons \mathrm{A}^{q+} \\ \mathrm{A}^{q+} + q\,\mathrm{e}^- &\longrightarrow \mathrm{A}. \end{aligned}$$

The overall current is determined by the rate of the RDS. Assuming Butler-Volmer kinetics for the forward rate constant of the RDS, we have,

$$\text{rate} = k_0 \left\{ a_{\mathrm{A}^{q+}} \exp\left[-\beta q e (1-\alpha) \cdot (\phi - \phi_{\text{eq.}})\right] - a_{\mathrm{A}} \exp\left[+\beta q e \alpha \cdot (\phi - \phi_{\text{eq.}})\right] \right\}, \tag{12}$$

where k_0 is the rate prefactor (sometimes called the Arrhenius prefactor), a_i is the activity of species i, $\beta \equiv (k_\mathrm{B} T)^{-1}$ is the inverse thermodynamic temperature, e is the fundamental charge, α is the symmetry coefficient, ϕ is the applied potential, and $\phi_{\text{eq.}}$ is the equilibrium potential for the RDS. At sufficiently high reductive overpotentials $\phi - \phi_{\text{eq.}} \ll 0$, only the first term survives,

$$\text{rate} \approx k_0 a_{\mathrm{A}^{q+}} \exp\left[-\beta q e (1-\alpha) \cdot (\phi - \phi_{\text{eq.}})\right]. \tag{13}$$

To make further progress, we have to solve for the activity of the intermediate species A^{q+} in terms of the activity of the reactant species for the overall reaction, $A^{(n+q)+}$. If we assume

that all steps prior to the RDS are fast and equilibriated, we can extract this activity by analyzing the thermodynamics of the steps prior to the RDS. The free energy change associated with the r'th reaction reads,

$$\Delta F_r = -\beta^{-1} \log a_{\mathrm{A}^{(n+q-(r+1))+}} + \beta^{-1} \log a_{\mathrm{A}^{(n+q-r)+}} + e\phi. \tag{14}$$

Then, the equilibrium constant for reaction r goes as,

$$K_r \equiv \exp\left[-\beta \Delta F_r\right] \tag{15}$$

$$K_r = \frac{a_{\mathrm{A}^{(n+q-(r+1))+}}}{a_{\mathrm{A}^{(n+q-r)+}}} \times \exp\left[-\beta e\phi\right] \tag{16}$$

$$K_r = \tilde{K}_r \times \exp\left[-\beta e\phi\right], \tag{17}$$

where $\tilde{K}_r$ is defined by Eq. (17), and is independent of potential. Since Eq. (17) holds for *all* reactions r before the RDS, we can easily solve for the activity of the intermediate,

$$a_{\mathrm{A}^{q+}} = \left[\prod_{r=1}^{n} \tilde{K}_r\right] \exp\left[-n\beta e\phi\right] \times a_{\mathrm{A}^{(n+q)+}} \tag{18}$$

$$a_{\mathrm{A}^{q+}} = \left[\prod_{r=1}^{n} \tilde{K}_r\right] \exp\left[-n\beta e\left(\phi - \phi_{\mathrm{eq.}}\right)\right] \exp\left[-n\beta e\phi_{\mathrm{eq.}}\right] \times a_{\mathrm{A}^{(n+q)+}}. \tag{19}$$

Plugging back into Eq. (13),

$$\text{rate} \approx k_0 \left(\left[\prod_{r=1}^{n} \tilde{K}_r\right] \exp\left[-n\beta e\left(\phi - \phi_{\mathrm{eq.}}\right)\right] \exp\left[-n\beta e\phi_{\mathrm{eq.}}\right] \times a_{\mathrm{A}^{(n+q)+}} \right) \exp\left[-\beta q e(1-\alpha) \cdot \left(\phi - \phi_{\mathrm{eq.}}\right)\right] \tag{20}$$

$$\text{rate} = k_0 \left(\left[\prod_{r=1}^{n} \tilde{K}_r\right] \exp\left[-n\beta e\phi_{\mathrm{eq.}}\right] \times a_{\mathrm{A}^{(n+q)+}} \right) \exp\left[-\beta e\left(\phi - \phi_{\mathrm{eq.}}\right)\left(n + q\cdot(1-\alpha)\right)\right]. \tag{21}$$

This is a mess, but we only care about the potential-dependent terms when extracting the Tafel slope, which means we only have to consider the last factor on the RHS. Taking the

logarithm yields,

$$\log\left[\text{rate}\right] = -\beta e\left(\phi - \phi_{\text{eq.}}\right)\left(n + q \cdot (1-\alpha)\right) + C, \tag{22}$$

where C is a constant independent of potential. For a reduction reaction, the Tafel slope is defined as,

$$\text{Tafel Slope} = \left[\frac{\partial \log_{10}\left[\text{rate}\right]}{\partial\left[-\left(\phi - \phi_{\text{eq.}}\right)\right]}\right]^{-1}. \tag{23}$$

Hence, we have,

$$\text{Tafel Slope} = \left[\log_{10}\left(\exp(1)\right) \cdot \beta e\right]^{-1} \cdot \frac{1}{n + q \cdot (1-\alpha)}. \tag{24}$$

Appropriate unit scalings yield,

$$\text{Tafel Slope} = \frac{60 \text{ mV/decade}}{n + q \cdot (1-\alpha)}, \tag{25}$$

which reduces to the equation quoted in the main text when $\alpha = 1/2$.

Physical Non-Idealities

Tafel Slopes with Physical Non-Idealities

Eq. (25) already accounts for the non-ideality effects introduced by $\alpha \neq 1/2$. If we assume that the CO_2 adsorption step has partial charge transfer character quantified by γ, then despite the fact that the adsorption step is purely chemical, we assume that its equilibrium constant carries a non-integer order dependence on the applied potential. This can also be motivated by considering the formation of a permanent dipole on the surface species, which can access additional thermodynamic stabilization due to a dipole Stark shift from electric fields present at theinterface. The manner in which surface dipole formation augments the Tafel slope depends on whether or not the CO_2 adsorption step is the rate determining step. For the case $(n, q) = (0, 1)$, the adsorption step is the RDS. The Frumkin correction

attenuates the applied potential for the rate-determining step by a factor f, which simply multiplies the latter term in the denominator of Eq. (25). Hence, the Tafel slope for $(n, q) = (0, 1)$ is,

$$\text{Tafel Slope} = \frac{60 \text{ mV/decade}}{\gamma \cdot f \cdot (1-\alpha)}. \tag{26}$$

For the case $(n, q) = (1, 0)$, the adsorption step is the RDS, and rather than contributing $n = 1$ to the order, it instead contributes according to the γ parameter,

$$\text{Tafel Slope} = \frac{60 \text{ mV/decade}}{\gamma}. \tag{27}$$

Finally, for the case $(n, q) = (1, 1)$, the adsorption step occurs before the RDS, and the Tafel slope reads,

$$\text{Tafel Slope} = \frac{60 \text{ mV/decade}}{\gamma + f \cdot (1-\alpha)}. \tag{28}$$

Sensitivities to Parameter Bounds

Figures 13 and 14 study the sensitivity of the distributional results presented in Fig. 4 of the main text to the bounds of the uniform distributions over non-ideality parameters. Broadly, our claim is supported by the sensitivity analysis; within reasonable parameter bounds, we still see that we can get essentially arbitrary distributional shapes depending on the nonidealities included in the model.

Multiple Kinetic Regimes

Here, we examine the consequences of fitting current-voltage data from a system exhibiting multiple kinetic regimes to a model that only allows a single Tafel slope (as in Eq. (2) in the main text) by analyzing synthetic data. The synthetic data is generated from the model,

$$\frac{1}{i(E)} = \frac{1}{i_{\text{lim}}} + \frac{1 + \exp\left(-e\left[k_{\text{B}}T\right]^{-1} \times \left[E - E_{\text{eq},1}\right]\right)}{\exp\left(e\left[k_{\text{B}}T\right]^{-1} \times \alpha\left[E - E_{\text{eq},2}\right]\right)}, \tag{29}$$

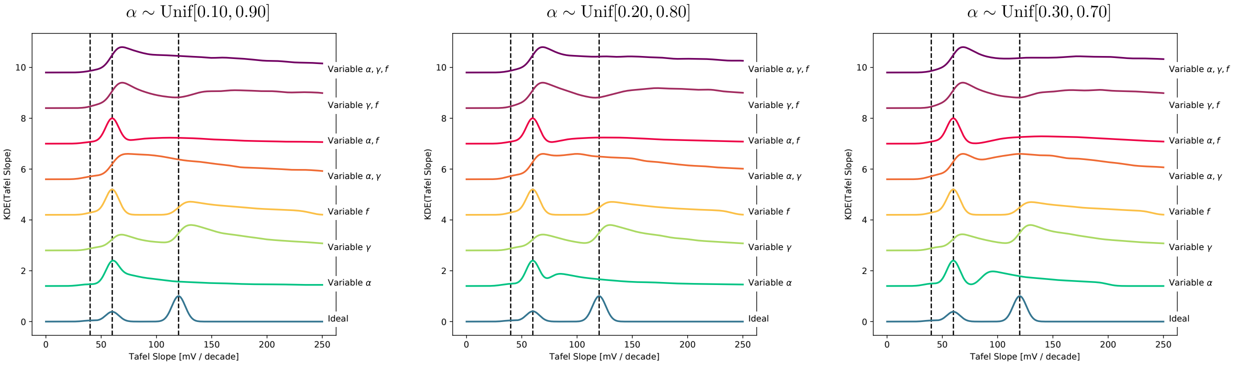

Supplementary Figure 13: Several synthetic kernel density estimates of the probability distributions over the Tafel slope generated from including random values of different parameters governing physical non-idealities. Different panels use different uniform distributions over the symmetry coefficient parameter, α.

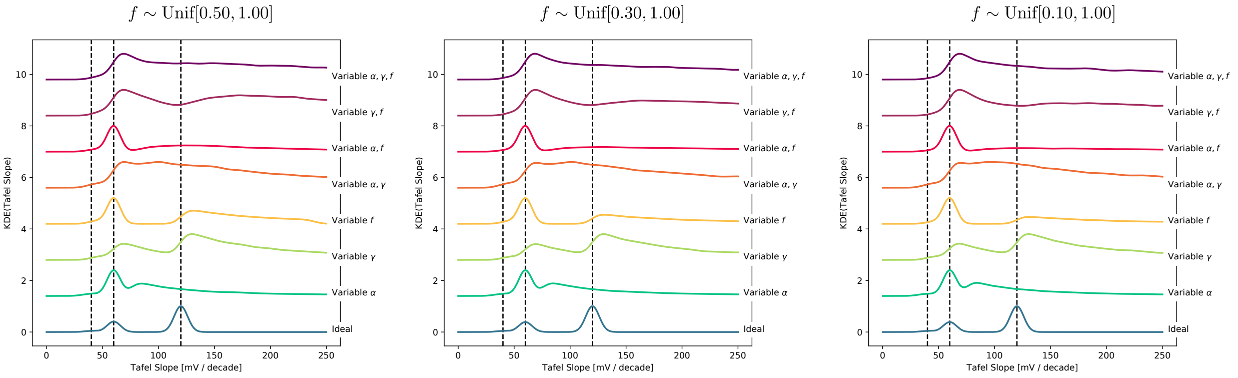

Supplementary Figure 14: Several synthetic kernel density estimates of the probability distributions over the Tafel slope generated from including random values of different parameters governing physical non-idealities. Different panels use different uniform distributions over the Frumkin correction parameter, f.

where E is the applied potential, and the model has free fitting parameters α, i_{lim}, $E_{\mathrm{eq},1}$, and $E_{\mathrm{eq},2}$. This model can be shown to arise when a reaction proceeds through a rate-limiting surface reaction involving a surface intermediate generated through a one-electron transfer, and present at non-negligible surface coverages. The specifics of how this model arises are less relevant to this analysis than the fact that the model exhibits two different Tafel regimes. When $\alpha = 1/2$, the first regime has Tafel slope $m_{\mathrm{T}} = 40$ mV decade^{-1} for $E_{\mathrm{eq},1} < E < E_{\mathrm{eq},2}$, and the second regime has a Tafel slope $m_{\mathrm{T}} = 120$ mV decade^{-1} for $E > E_{\mathrm{eq},2}$, before topping out at the limiting current i_{lim}.

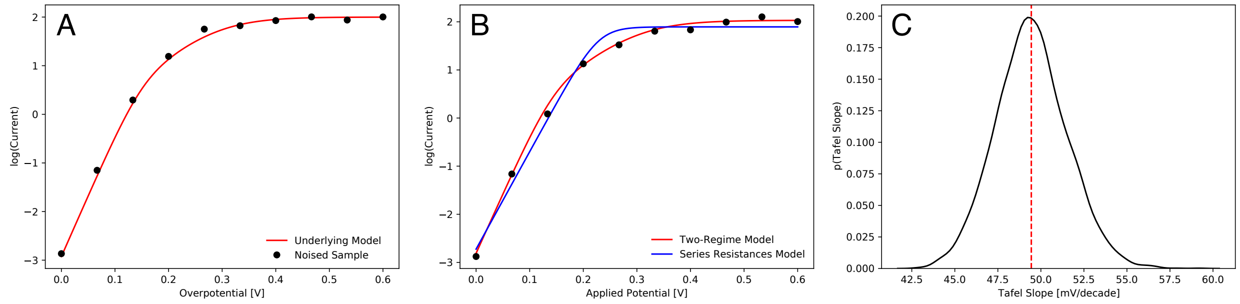

Supplementary Figure 15: (A) Current-voltage trace generated from Eq. (29) using the parameters $\alpha = 1/2$, $i_{\mathrm{lim}} = 100$ mA cm^{-2}, $E_{\mathrm{eq},1} = 0.05$ V, and $E_{\mathrm{eq},2} = 0.15$ V (red trace), along with artificially noised data sampled from the model (black points). (B) Current-voltage traces evaluated from the MAP fit parameters for Eq. (2) in the main text and Eq. (29), along with the noised data used for the fits (black points). (C) Posterior distribution over the Tafel slope for the model described by Eq. (2) in the main text.

Figure 15A shows a trace of the current-voltage behavior predicted by Eq. (29), with parameters $\alpha = 1/2$, $i_{\mathrm{lim}} = 100$ mA cm^{-2}, $E_{\mathrm{eq},1} = 0.05$ V, and $E_{\mathrm{eq},2} = 0.15$ V, as well as artificially noised data sampled from this model at a sparsely sampled set of voltage points. Figure 15B shows current-voltage traces from both Eq. (29) (red) and Eq. (2) from the main text (blue), each evaluated under the MAP parameters determined from their respective Bayesian fits. Finally, Fig. 15C shows the Bayes posterior distribution for the Tafel slope fitted using Eq. (2) from the main text. As expected, when using the single Tafel regime model to fit data generated from multiple Tafel regimes, the MAP value of the Tafel slope does not coincide with the Tafel slope from *either* kinetic regime in Eq. (29).

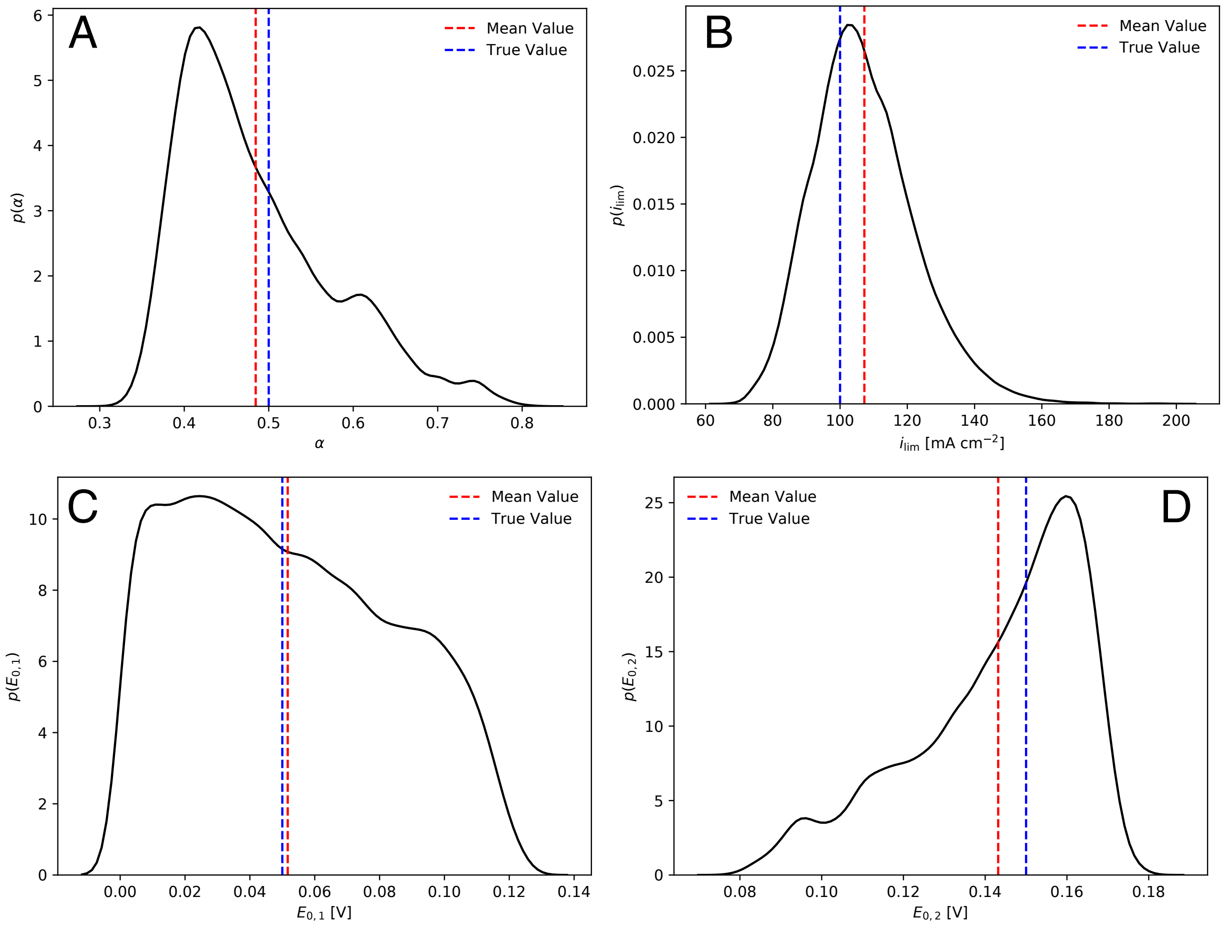

Supplementary Figure 16: Posterior distributions for α, $i_{\rm lim}$, $E_{\rm eq,1}$, and $E_{\rm eq,2}$ (A, B, C, D, respectively) gleaned from Bayesian posterior sampling on the artificially noised data in Fig. 15A.

However, as illustrated in Fig. 16, when the original data is fit to Eq. (29), the posterior distributions are peaked around the true values of the parameters.

This analysis yields two important takeaways. First, it lends credence to the idea that fitting a single Tafel slope to data collected under multiple Tafel regimes, each individually exhibiting a cardinal Tafel slope, can produce an off-cardinal value of the Tafel slope, providing an alternative possible explanation for the lack of observed cardinality in the literature analysis in the main text. Second, and perhaps more importantly, it shows that the Bayesian framework presented here can successfully estimate the parameters of more complicated physical models that incorporate the effects of multiple different Tafel regimes, or some of the physical nonidealities discussed in the main text. Practically, we furnish the following recommendation: if one knows in advance that multiple kinetic regimes are at play in a set of current-voltage data, and the nature of these regimes can be encoded into a kinetic model like Eq. (29), then one should carry out Bayesian fitting to such a model. In the absence of sufficient independent evidence to pin down a kinetic model that resolves multiple kinetic regimes, Eq. (2) from the main text is a viable alternative, but one should be very cautious about over-interpreting the mechanistic implications of a Tafel slope determined in this manner.

References

(1) Manthiram, K.; Beberwyck, B. J.; Alivisatos, A. P. Enhanced Electrochemical Methanation of Carbon Dioxide with a Dispersible Nanoscale Copper Catalyst. *J. Am. Chem. Soc. 136*, 13319–13325, DOI: 10.1021/ja5065284, (2014).

(2) Chen, Y.; Li, C. W.; Kanan, M. W. Aqueous CO_2 Reduction at Very Low Overpotential on Oxide-Derived Au Nanoparticles. *J. Am. Chem. Soc. 134*, 19969–19972, DOI: 10.1021/ja309317u, (2012).

(3) Noda, H.; Ikeda, S.; Yamamoto, A.; Einaga, H.; Ito, K. Kinetics of Electrochemical Reduction of Carbon Dioxide on a Gold Electrode in Phosphate Buffer Solutions. *Bull. Chem. Soc. Jpn. 68*, 1889–1895, DOI: 10.1246/bcsj.68.1889, (1995).

(4) Ma, M.; Trześniewski, B. J.; Xie, J.; Smith, W. A. Selective and Efficient Reduction of Carbon Dioxide to Carbon Monoxide on Oxide-Derived Nanostructured Silver Electrocatalysts. *Angew. Chem. Int. Ed. 55*, 9748–9752, DOI: 10.1002/anie.201604654, (2016).

(5) Wu, J.; Yadav, R. M.; Liu, M.; Sharma, P. P.; Tiwary, C. S.; Ma, L.; Zou, X.; Zhou, X.-D.; Yakobson, B. I.; Lou, J.; Ajayan, P. M. Achieving Highly Efficient, Selective, and Stable CO_2 Reduction on Nitrogen-Doped Carbon Nanotubes. *ACS Nano 9*, 5364–5371, DOI: 10.1021/acsnano.5b01079, (2015).

(6) Gu, J.; Héroguel, F.; Luterbacher, J.; Hu, X. Densely Packed, Ultra Small SnO Nanoparticles for Enhanced Activity and Selectivity in Electrochemical CO_2 Reduction. *Angew. Chem. Int. Ed. 57*, 2943–2947, DOI: 10.1002/anie.201713003, (2018).

(7) Li, X.; Bi, W.; Chen, M.; Sun, Y.; Ju, H.; Yan, W.; Zhu, J.; Wu, X.; Chu, W.; Wu, C.; Xie, Y. Exclusive Ni–N_4 Sites Realize Near-Unity CO Selectivity for Electrochemical CO_2 Reduction. *J. Am. Chem. Soc. 139*, 14889–14892, DOI: 10.1021/jacs.7b09074, (2017).

(8) Zhu, M.; Chen, J.; Huang, L.; Ye, R.; Xu, J.; Han, Y. Covalently Grafting Cobalt Porphyrin onto Carbon Nanotubes for Efficient CO $_2$ Electroreduction. *Angew. Chem. Int. Ed. 58*, 6595–6599, DOI: 10.1002/anie.201900499, (2019).

(9) Wang, Y.; Liu, J.; Wang, Y.; Al-Enizi, A. M.; Zheng, G. Tuning of CO $_2$ Reduction Selectivity on Metal Electrocatalysts. *Small 13*, 1701809, DOI: 10.1002/smll.201701809, (2017).

(10) Ma, T.; Fan, Q.; Tao, H.; Han, Z.; Jia, M.; Gao, Y.; Ma, W.; Sun, Z. Heterogeneous electrochemical CO $_2$ reduction using nonmetallic carbon-based catalysts: current status and future challenges. *Nanotechnology 28*, 472001, DOI: 10.1088/1361-6528/aa8f6f, (2017).

(11) Liu, Y.; Chen, S.; Quan, X.; Yu, H. Efficient Electrochemical Reduction of Carbon Dioxide to Acetate on Nitrogen-Doped Nanodiamond. *J. Am. Chem. Soc. 137*, 11631–11636, DOI: 10.1021/jacs.5b02975, (2015).

(12) Su, P.; Iwase, K.; Nakanishi, S.; Hashimoto, K.; Kamiya, K. Nickel-Nitrogen-Modified Graphene: An Efficient Electrocatalyst for the Reduction of Carbon Dioxide to Carbon Monoxide. *Small 12*, 6083–6089, DOI: 10.1002/smll.201602158, (2016).

(13) Shao, P.; Ci, S.; Yi, L.; Cai, P.; Huang, P.; Cao, C.; Wen, Z. Hollow CuS Microcube Electrocatalysts for CO $_2$ Reduction Reaction. *ChemElectroChem 4*, 2593–2598, DOI: 10.1002/celc.201700517, (2017).

(14) Xu, J.; Kan, Y.; Huang, R.; Zhang, B.; Wang, B.; Wu, K.-H.; Lin, Y.; Sun, X.; Li, Q.; Centi, G.; Su, D. Revealing the Origin of Activity in Nitrogen-Doped Nanocarbons towards Electrocatalytic Reduction of Carbon Dioxide. *ChemSusChem 9*, 1085–1089, DOI: 10.1002/cssc.201600202, (2016).

(15) Medina-Ramos, J.; DiMeglio, J. L.; Rosenthal, J. Efficient Reduction of CO $_2$ to CO

with High Current Density Using in Situ or ex Situ Prepared Bi-Based Materials. *J. Am. Chem. Soc. 136*, 8361–8367, DOI: 10.1021/ja501923g, (2014).

(16) Ma, M.; Liu, K.; Shen, J.; Kas, R.; Smith, W. A. In Situ Fabrication and Reactivation of Highly Selective and Stable Ag Catalysts for Electrochemical CO $_2$ Conversion. *ACS Energy Lett. 3*, 1301–1306, DOI: 10.1021/acsenergylett.8b00472, (2018).

(17) Zhang, S.; Kang, P.; Meyer, T. J. Nanostructured Tin Catalysts for Selective Electrochemical Reduction of Carbon Dioxide to Formate. *J. Am. Chem. Soc. 136*, 1734–1737, DOI: 10.1021/ja4113885, (2014).

(18) Jiang, X.; Cai, F.; Gao, D.; Dong, J.; Miao, S.; Wang, G.; Bao, X. Electrocatalytic reduction of carbon dioxide over reduced nanoporous zinc oxide. *Electrochem. Commun. 68*, 67–70, DOI: 10.1016/j.elecom.2016.05.003, (2016).

(19) Chen, Y.; Kanan, M. W. Tin Oxide Dependence of the CO $_2$ Reduction Efficiency on Tin Electrodes and Enhanced Activity for Tin/Tin Oxide Thin-Film Catalysts. *J. Am. Chem. Soc. 134*, 1986–1989, DOI: 10.1021/ja2108799, (2012).

(20) Chang, Z.; Huo, S.; Zhang, W.; Fang, J.; Wang, H. The Tunable and Highly Selective Reduction Products on Ag@Cu Bimetallic Catalysts Toward CO $_2$ Electrochemical Reduction Reaction. *J. Phys. Chem. C 121*, 11368–11379, DOI: 10.1021/acs.jpcc.7b01586, (2017).

(21) Koh, J. H.; Won, D. H.; Eom, T.; Kim, N.-K.; Jung, K. D.; Kim, H.; Hwang, Y. J.; Min, B. K. Facile CO $_2$ Electro-Reduction to Formate via Oxygen Bidentate Intermediate Stabilized by High-Index Planes of Bi Dendrite Catalyst. *ACS Catal. 7*, 5071–5077, DOI: 10.1021/acscatal.7b00707, (2017).

(22) Jianping, Q.; Juntao, T.; Jie, S.; Cuiwei, W.; Mengqian, Q.; Zhiqiao, H.; Jianmeng, C.; Song, S. Preparation of a silver electrode with a three-dimensional surface and its

performance in the electrochemical reduction of carbon dioxide. *Electrochim. Acta 203*, 99–108, DOI: 10.1016/j.electacta.2016.03.182, (2016).

(23) Qiu, Y.; Du, J.; Dong, W.; Dai, C.; Tao, C. Selective conversion of CO 2 to formate on a size tunable nano-Bi electrocatalyst. *J. CO2 Util. 20*, 328–335, DOI: 10.1016/j.jcou.2017.05.024, (2017).

(24) Karapinar, D.; Zitolo, A.; Huan, T. N.; Zanna, S.; Taverna, D.; Galvão Tizei, L. H.; Giaume, D.; Marcus, P.; Mougel, V.; Fontecave, M. Carbon-Nanotube-Supported Copper Polyphthalocyanine for Efficient and Selective Electrocatalytic CO_2 Reduction to CO. *ChemSusChem 13*, 173–179, DOI: 10.1002/cssc.201902859, (2020).

(25) Luan, C.; Shao, Y.; Lu, Q.; Gao, S.; Huang, K.; Wu, H.; Yao, K. High-Performance Carbon Dioxide Electrocatalytic Reduction by Easily Fabricated Large-Scale Silver Nanowire Arrays. *ACS Appl. Mater. Interfaces 10*, 17950–17956, DOI: 10.1021/acsami.8b03461, (2018).

(26) Peng, Y.; Wu, T.; Sun, L.; Nsanzimana, J. M. V.; Fisher, A. C.; Wang, X. Selective Electrochemical Reduction of CO_2 to Ethylene on Nanopores-Modified Copper Electrodes in Aqueous Solution. *ACS Appl. Mater. Interfaces 9*, 32782–32789, DOI: 10.1021/acsami.7b10421, (2017).

(27) Lu, H.; Zhang, L.; Zhong, J. H.; Yang, H. G. Partially Oxidized Palladium Nanodots for Enhanced Electrocatalytic Carbon Dioxide Reduction. *Chem. Asian J. 13*, 2800–2804, DOI: 10.1002/asia.201800946, (2018).

(28) Guo, S.; Zhang, Y.; Zhang, X.; Easton, C. D.; MacFarlane, D. R.; Zhang, J. Phosphomolybdic Acid-Assisted Growth of Ultrathin Bismuth Nanosheets for Enhanced Electrocatalytic Reduction of CO_2 to Formate. *ChemSusChem 12*, 1091–1100, DOI: 10.1002/cssc.201802409, (2019).

(29) Raciti, D.; Wang, Y.; Park, J. H.; Wang, C. Three-Dimensional Hierarchical Copper-Based Nanostructures as Advanced Electrocatalysts for CO $_2$ Reduction. *ACS Appl. Energy Mater. 1*, 2392–2398, DOI: 10.1021/acsaem.8b00356, (2018).

(30) Kauffman, D. R.; Alfonso, D. R.; Tafen, D. N.; Wang, C.; Zhou, Y.; Yu, Y.; Lekse, J. W.; Deng, X.; Espinoza, V.; Trindell, J.; Ranasingha, O. K.; Roy, A.; Lee, J.-S.; Xin, H. L. Selective Electrocatalytic Reduction of CO $_2$ into CO at Small, Thiol-Capped Au/Cu Nanoparticles. *J. Phys. Chem. C 122*, 27991–28000, DOI: 10.1021/acs.jpcc.8b06234, (2018).

(31) Bejtka, K.; Zeng, J.; Sacco, A.; Castellino, M.; Hernández, S.; Farkhondehfal, M. A.; Savino, U.; Ansaloni, S.; Pirri, C. F.; Chiodoni, A. Chainlike Mesoporous SnO $_2$ as a Well-Performing Catalyst for Electrochemical CO $_2$ Reduction. *ACS Appl. Energy Mater. 2*, 3081–3091, DOI: 10.1021/acsaem.8b02048, (2019).

(32) Liu, H.; Xiang, K.; Liu, Y.; Zhu, F.; Zou, M.; Yan, X.; Chai, L. Polydopamine Functionalized Cu Nanowires for Enhanced CO $_2$ Electroreduction Towards Methane. *ChemElectroChem 5*, 3991–3999, DOI: 10.1002/celc.201801132, (2018).

(33) Duan, Y.-X.; Meng, F.-L.; Liu, K.-H.; Yi, S.-S.; Li, S.-J.; Yan, J.-M.; Jiang, Q. Amorphizing of Cu Nanoparticles toward Highly Efficient and Robust Electrocatalyst for CO $_2$ Reduction to Liquid Fuels with High Faradaic Efficiencies. *Adv. Mater. 30*, 1706194, DOI: 10.1002/adma.201706194, (2018).

(34) Lu, Q.; Rosen, J.; Zhou, Y.; Hutchings, G. S.; Kimmel, Y. C.; Chen, J. G.; Jiao, F. A selective and efficient electrocatalyst for carbon dioxide reduction. *Nat. Commun. 5*, 3242, DOI: 10.1038/ncomms4242, (2014).

(35) Hsieh, Y.-C.; Senanayake, S. D.; Zhang, Y.; Xu, W.; Polyansky, D. E. Effect of Chloride Anions on the Synthesis and Enhanced Catalytic Activity of Silver

Nanocoral Electrodes for CO_2 Electroreduction. *ACS Catal. 5*, 5349–5356, DOI: 10.1021/acscatal.5b01235, (2015).

(36) Cui, X.; Pan, Z.; Zhang, L.; Peng, H.; Zheng, G. Selective Etching of Nitrogen-Doped Carbon by Steam for Enhanced Electrochemical CO_2 Reduction. *Adv. Energy Mater. 7*, 1701456, DOI: 10.1002/aenm.201701456, (2017).

(37) Han, N. et al. Supported Cobalt Polyphthalocyanine for High-Performance Electrocatalytic CO2 Reduction. *Chem 3*, 652–664, DOI: 10.1016/j.chempr.2017.08.002, (2017).

(38) Li, F.; Chen, L.; Knowles, G. P.; MacFarlane, D. R.; Zhang, J. Hierarchical Mesoporous SnO_2 Nanosheets on Carbon Cloth: A Robust and Flexible Electrocatalyst for CO_2 Reduction with High Efficiency and Selectivity. *Angew. Chem. Int. Ed. 56*, 505–509, DOI: 10.1002/anie.201608279, (2017).

(39) Pan, F.; Deng, W.; Justiniano, C.; Li, Y. Identification of champion transition metals centers in metal and nitrogen-codoped carbon catalysts for CO2 reduction. *Appl. Catal. B 226*, 463–472, DOI: 10.1016/j.apcatb.2018.01.001, (2018).

(40) Li, C. W.; Kanan, M. W. CO_2 Reduction at Low Overpotential on Cu Electrodes Resulting from the Reduction of Thick Cu_2O Films. *J. Am. Chem. Soc. 134*, 7231–7234, DOI: 10.1021/ja3010978, (2012).

(41) Lu, P.; Yang, Y.; Yao, J.; Wang, M.; Dipazir, S.; Yuan, M.; Zhang, J.; Wang, X.; Xie, Z.; Zhang, G. Facile synthesis of single-nickel-atomic dispersed N-doped carbon framework for efficient electrochemical CO2 reduction. *Appl. Catal. B 241*, 113–119, DOI: 10.1016/j.apcatb.2018.09.025, (2019).

(42) Luc, W.; Collins, C.; Wang, S.; Xin, H.; He, K.; Kang, Y.; Jiao, F. Ag–Sn Bimetallic Catalyst with a Core–Shell Structure for CO_2 Reduction. *J. Am. Chem. Soc. 139*, 1885–1893, DOI: 10.1021/jacs.6b10435, (2017).

(43) Rogers, C.; Perkins, W. S.; Veber, G.; Williams, T. E.; Cloke, R. R.; Fischer, F. R. Synergistic Enhancement of Electrocatalytic CO $_2$ Reduction with Gold Nanoparticles Embedded in Functional Graphene Nanoribbon Composite Electrodes. *J. Am. Chem. Soc. 139*, 4052–4061, DOI: 10.1021/jacs.6b12217, (2017).

(44) Cao, Z.; Zacate, S. B.; Sun, X.; Liu, J.; Hale, E. M.; Carson, W. P.; Tyndall, S. B.; Xu, J.; Liu, X.; Liu, X.; Song, C.; Luo, J.-h.; Cheng, M.-J.; Wen, X.; Liu, W. Tuning Gold Nanoparticles with Chelating Ligands for Highly Efficient Electrocatalytic CO $_2$ Reduction. *Angew. Chem. 130*, 12857–12861, DOI: 10.1002/ange.201805696, (2018).

(45) School of Petrochemical Engineering, Changzhou University, Changzhou 213164, P. R. China,; Bei, J. Efficient Reduction of CO2 to Formate Using in Situ Prepared Nano-Sized Bi Electrocatalyst. *Int. J. Electrochem. Sci.* 2365–2375, DOI: 10.20964/2017.03.72, (2017).

(46) Su, P.; Xu, W.; Qiu, Y.; Zhang, T.; Li, X.; Zhang, H. Ultrathin Bismuth Nanosheets as a Highly Efficient CO $_2$ Reduction Electrocatalyst. *ChemSusChem 11*, 848–853, DOI: 10.1002/cssc.201702229, (2018).

(47) Yang, H.; Han, N.; Deng, J.; Wu, J.; Wang, Y.; Hu, Y.; Ding, P.; Li, Y.; Li, Y.; Lu, J. Selective CO $_2$ Reduction on 2D Mesoporous Bi Nanosheets. *Adv. Energy Mater. 8*, 1801536, DOI: 10.1002/aenm.201801536, (2018).

(48) Han, P.; Yu, X.; Yuan, D.; Kuang, M.; Wang, Y.; Al-Enizi, A. M.; Zheng, G. Defective graphene for electrocatalytic CO2 reduction. *J. Colloid Interface Sci. 534*, 332–337, DOI: 10.1016/j.jcis.2018.09.036, (2019).

(49) Zheng, X.; Han, J.; Fu, Y.; Deng, Y.; Liu, Y.; Yang, Y.; Wang, T.; Zhang, L. Highly efficient CO2 reduction on ordered porous Cu electrode derived from Cu2O inverse opals. *Nano Energy 48*, 93–100, DOI: 10.1016/j.nanoen.2018.03.023, (2018).

(50) Jiang, B.; Zhang, X.-G.; Jiang, K.; Wu, D.-Y.; Cai, W.-B. Boosting Formate Production in Electrocatalytic CO_2 Reduction over Wide Potential Window on Pd Surfaces. *J. Am. Chem. Soc. 140*, 2880–2889, DOI: 10.1021/jacs.7b12506, (2018).

(51) Rosen, J.; Hutchings, G. S.; Lu, Q.; Forest, R. V.; Moore, A.; Jiao, F. Electrodeposited Zn Dendrites with Enhanced CO Selectivity for Electrocatalytic CO_2 Reduction. *ACS Catal. 5*, 4586–4591, DOI: 10.1021/acscatal.5b00922, (2015).

(52) Jiang, K.; Wang, H.; Cai, W.-B.; Wang, H. Li Electrochemical Tuning of Metal Oxide for Highly Selective CO_2 Reduction. *ACS Nano 11*, 6451–6458, DOI: 10.1021/acsnano.7b03029, (2017).

(53) Cho, M.; Song, J. T.; Back, S.; Jung, Y.; Oh, J. The Role of Adsorbed CN and Cl on an Au Electrode for Electrochemical CO_2 Reduction. *ACS Catal. 8*, 1178–1185, DOI: 10.1021/acscatal.7b03449, (2018).

(54) Lv, W.; Zhou, J.; Bei, J.; Zhang, R.; Wang, L.; Xu, Q.; Wang, W. Electrodeposition of nano-sized bismuth on copper foil as electrocatalyst for reduction of CO2 to formate. *Appl. Surf. Sci. 393*, 191–196, DOI: 10.1016/j.apsusc.2016.10.017, (2017).

(55) Xie, J.; Zhao, X.; Wu, M.; Li, Q.; Wang, Y.; Yao, J. Metal-Free Fluorine-Doped Carbon Electrocatalyst for CO_2 Reduction Outcompeting Hydrogen Evolution. *Angew. Chem. Int. Ed. 57*, 9640–9644, DOI: 10.1002/anie.201802055, (2018).

(56) Pan, F.; Zhao, H.; Deng, W.; Feng, X.; Li, Y. A novel N,Fe-Decorated carbon nanotube/carbon nanosheet architecture for efficient CO2 reduction. *Electrochim. Acta 273*, 154–161, DOI: 10.1016/j.electacta.2018.04.047, (2018).

(57) Kornienko, N.; Zhao, Y.; Kley, C. S.; Zhu, C.; Kim, D.; Lin, S.; Chang, C. J.; Yaghi, O. M.; Yang, P. Metal–Organic Frameworks for Electrocatalytic Reduction of Carbon Dioxide. *J. Am. Chem. Soc. 137*, 14129–14135, DOI: 10.1021/jacs.5b08212, (2015).

(58) Yang, H.; Wu, Y.; Lin, Q.; Fan, L.; Chai, X.; Zhang, Q.; Liu, J.; He, C.; Lin, Z. Composition Tailoring via N and S Co-doping and Structure Tuning by Constructing Hierarchical Pores: Metal-Free Catalysts for High-Performance Electrochemical Reduction of CO $_2$. *Angew. Chem. Int. Ed. 57*, 15476–15480, DOI: 10.1002/anie.201809255, (2018).

(59) Geng, Z.; Kong, X.; Chen, W.; Su, H.; Liu, Y.; Cai, F.; Wang, G.; Zeng, J. Oxygen Vacancies in ZnO Nanosheets Enhance CO $_2$ Electrochemical Reduction to CO. *Angew. Chem. Int. Ed. 57*, 6054–6059, DOI: 10.1002/anie.201711255, (2018).

(60) Zhang, W.; Hu, Y.; Ma, L.; Zhu, G.; Zhao, P.; Xue, X.; Chen, R.; Yang, S.; Ma, J.; Liu, J.; Jin, Z. Liquid-phase exfoliated ultrathin Bi nanosheets: Uncovering the origins of enhanced electrocatalytic CO2 reduction on two-dimensional metal nanostructure. *Nano Energy 53*, 808–816, DOI: 10.1016/j.nanoen.2018.09.053, (2018).

(61) Cao, Z.; Derrick, J. S.; Xu, J.; Gao, R.; Gong, M.; Nichols, E. M.; Smith, P. T.; Liu, X.; Wen, X.; Copéret, C.; Chang, C. J. Chelating N-Heterocyclic Carbene Ligands Enable Tuning of Electrocatalytic CO $_2$ Reduction to Formate and Carbon Monoxide: Surface Organometallic Chemistry. *Angew. Chem. Int. Ed. 57*, 4981–4985, DOI: 10.1002/anie.201800367, (2018).

(62) Zhao, C.; Wang, J. Electrochemical reduction of CO 2 to formate in aqueous solution using electro-deposited Sn catalysts. *Chem. Eng. J. 293*, 161–170, DOI: 10.1016/j.cej.2016.02.084, (2016).

(63) Zhang, X.; Lei, T.; Liu, Y.; Qiao, J. Enhancing CO2 electrolysis to formate on facilely synthesized Bi catalysts at low overpotential. *Appl. Catal. B 218*, 46–50, DOI: 10.1016/j.apcatb.2017.06.032, (2017).

(64) He, J.; Liu, X.; Liu, H.; Zhao, Z.; Ding, Y.; Luo, J. Highly selective electrocatalytic

reduction of CO2 to formate over Tin(IV) sulfide monolayers. *J. Catal. 364*, 125–130, DOI: 10.1016/j.jcat.2018.05.005, (2018).

(65) Smith, P. T.; Benke, B. P.; Cao, Z.; Kim, Y.; Nichols, E. M.; Kim, K.; Chang, C. J. Iron Porphyrins Embedded into a Supramolecular Porous Organic Cage for Electrochemical CO $_2$ Reduction in Water. *Angew. Chem. Int. Ed. 57*, 9684–9688, DOI: 10.1002/anie.201803873, (2018).

(66) Zhang, A.; He, R.; Li, H.; Chen, Y.; Kong, T.; Li, K.; Ju, H.; Zhu, J.; Zhu, W.; Zeng, J. Nickel Doping in Atomically Thin Tin Disulfide Nanosheets Enables Highly Efficient CO $_2$ Reduction. *Angew. Chem. Int. Ed. 57*, 10954–10958, DOI: 10.1002/anie.201806043, (2018).

(67) Morimoto, M.; Takatsuji, Y.; Hirata, K.; Fukuma, T.; Ohno, T.; Sakakura, T.; Haruyama, T. Visualization of catalytic edge reactivity in electrochemical CO2 reduction on porous Zn electrode. *Electrochim. Acta 290*, 255–261, DOI: 10.1016/j.electacta.2018.09.080, (2018).

(68) Spataru, N.; Tokuhiro, K.; Terashima, C.; Rao, T. N.; Fujishima, A. Electrochemical reduction of carbon dioxide at ruthenium dioxide deposited on boron-doped diamond. *J. Appl. Electrochem. 33*, 1205–1210, DOI: 10.1023/B:JACH.0000003866.85015.b6, (2003).

(69) Hirota, K.; Tryk, D. A.; Yamamoto, T.; Hashimoto, K.; Okawa, M.; Fujishima, A. Photoelectrochemical Reduction of CO $_2$ in a High-Pressure CO $_2$ + Methanol Medium at p-Type Semiconductor Electrodes. *J. Phys. Chem. B 102*, 9834–9843, DOI: 10.1021/jp9822945, (1998).

(70) Liu, X.; Yang, H.; He, J.; Liu, H.; Song, L.; Li, L.; Luo, J. Highly Active, Durable Ultrathin MoTe $_2$ Layers for the Electroreduction of CO $_2$ to CH $_4$. *Small 14*, 1704049, DOI: 10.1002/smll.201704049, (2018).

(71) Li, F.; Chen, L.; Xue, M.; Williams, T.; Zhang, Y.; MacFarlane, D. R.; Zhang, J. Towards a better Sn: Efficient electrocatalytic reduction of CO 2 to formate by Sn/SnS 2 derived from SnS 2 nanosheets. *Nano Energy 31*, 270–277, DOI: 10.1016/j.nanoen.2016.11.004, (2017).

(72) Hu, X.-M.; Rønne, M. H.; Pedersen, S. U.; Skrydstrup, T.; Daasbjerg, K. Enhanced Catalytic Activity of Cobalt Porphyrin in CO_2 Electroreduction upon Immobilization on Carbon Materials. *Angew. Chem. Int. Ed. 56*, 6468–6472, DOI: 10.1002/anie.201701104, (2017).

(73) Liu, S.; Tao, H.; Zeng, L.; Liu, Q.; Xu, Z.; Liu, Q.; Luo, J.-L. Shape-Dependent Electrocatalytic Reduction of CO_2 to CO on Triangular Silver Nanoplates. *J. Am. Chem. Soc. 139*, 2160–2163, DOI: 10.1021/jacs.6b12103, (2017).

(74) Liu, S.; Xiao, J.; Lu, X. F.; Wang, J.; Wang, X.; Lou, X. W. D. Efficient Electrochemical Reduction of CO_2 to HCOOH over Sub-2 nm SnO_2 Quantum Wires with Exposed Grain Boundaries. *Angew. Chem. Int. Ed. 58*, 8499–8503, DOI: 10.1002/anie.201903613, (2019).

(75) Liu, S.; Yang, H.; Huang, X.; Liu, L.; Cai, W.; Gao, J.; Li, X.; Zhang, T.; Huang, Y.; Liu, B. Identifying Active Sites of Nitrogen-Doped Carbon Materials for the CO_2 Reduction Reaction. *Adv. Funct. Mater. 28*, 1800499, DOI: 10.1002/adfm.201800499, (2018).

(76) Nursanto, E. B.; Jeon, H. S.; Kim, C.; Jee, M. S.; Koh, J. H.; Hwang, Y. J.; Min, B. K. Gold catalyst reactivity for CO2 electro-reduction: From nano particle to layer. *Catal. Today 260*, 107–111, DOI: 10.1016/j.cattod.2015.05.017, (2016).

(77) Peng, X.; Karakalos, S. G.; Mustain, W. E. Preferentially Oriented Ag Nanocrystals with Extremely High Activity and Faradaic Efficiency for CO_2 Electro-

chemical Reduction to CO. *ACS Appl. Mater. Interfaces 10*, 1734–1742, DOI: 10.1021/acsami.7b16164, (2018).

(78) Zhu, W.; Zhang, L.; Yang, P.; Chang, X.; Dong, H.; Li, A.; Hu, C.; Huang, Z.; Zhao, Z.-J.; Gong, J. Morphological and Compositional Design of Pd-Cu Bimetallic Nanocatalysts with Controllable Product Selectivity toward CO_2 Electroreduction. *Small 14*, 1703314, DOI: 10.1002/smll.201703314, (2018).

(79) Geng, Z.; Cao, Y.; Chen, W.; Kong, X.; Liu, Y.; Yao, T.; Lin, Y. Regulating the coordination environment of Co single atoms for achieving efficient electrocatalytic activity in CO2 reduction. *Appl. Catal. B 240*, 234–240, DOI: 10.1016/j.apcatb.2018.08.075, (2019).

(80) Kim, D.; Kley, C. S.; Li, Y.; Yang, P. Copper nanoparticle ensembles for selective electroreduction of CO_2 to C_2–C_3 products. *Proc. Natl. Acad. Sci. 114*, 10560–10565, DOI: 10.1073/pnas.1711493114, (2017).

(81) Chen, Z.; Mou, K.; Yao, S.; Liu, L. Zinc-Coordinated Nitrogen-Codoped Graphene as an Efficient Catalyst for Selective Electrochemical Reduction of CO_2 to CO. *ChemSusChem 11*, 2944–2952, DOI: 10.1002/cssc.201800925, (2018).

(82) Kim, S. K.; Zhang, Y.-J.; Bergstrom, H.; Michalsky, R.; Peterson, A. Understanding the Low-Overpotential Production of CH_4 from CO_2 on Mo_2C Catalysts. *ACS Catal. 6*, 2003–2013, DOI: 10.1021/acscatal.5b02424, (2016).

(83) Zhu, M.; Ye, R.; Jin, K.; Lazouski, N.; Manthiram, K. Elucidating the Reactivity and Mechanism of CO_2 Electroreduction at Highly Dispersed Cobalt Phthalocyanine. *ACS Energy Lett. 3*, 1381–1386, DOI: 10.1021/acsenergylett.8b00519, (2018).

(84) Kumar, B.; Atla, V.; Brian, J. P.; Kumari, S.; Nguyen, T. Q.; Sunkara, M.; Spurgeon, J. M. Reduced SnO_2 Porous Nanowires with a High Density of Grain Boundaries

as Catalysts for Efficient Electrochemical CO_2-into-HCOOH Conversion. *Angew. Chem. Int. Ed. 56*, 3645–3649, DOI: 10.1002/anie.201612194, (2017).

(85) Wang, H.; Jia, J.; Song, P.; Wang, Q.; Li, D.; Min, S.; Qian, C.; Wang, L.; Li, Y. F.; Ma, C.; Wu, T.; Yuan, J.; Antonietti, M.; Ozin, G. A. Efficient Electrocatalytic Reduction of CO_2 by Nitrogen-Doped Nanoporous Carbon/Carbon Nanotube Membranes: A Step Towards the Electrochemical CO_2 Refinery. *Angew. Chem. Int. Ed. 56*, 7847–7852, DOI: 10.1002/anie.201703720, (2017).

(86) Wu, J.; Liu, M.; Sharma, P. P.; Yadav, R. M.; Ma, L.; Yang, Y.; Zou, X.; Zhou, X.-D.; Vajtai, R.; Yakobson, B. I.; Lou, J.; Ajayan, P. M. Incorporation of Nitrogen Defects for Efficient Reduction of CO_2 via Two-Electron Pathway on Three-Dimensional Graphene Foam. *Nano Lett. 16*, 466–470, DOI: 10.1021/acs.nanolett.5b04123, (2016).

(87) Ren, W.; Tan, X.; Yang, W.; Jia, C.; Xu, S.; Wang, K.; Smith, S. C.; Zhao, C. Isolated Diatomic Ni-Fe Metal–Nitrogen Sites for Synergistic Electroreduction of CO_2. *Angew. Chem. Int. Ed. 58*, 6972–6976, DOI: 10.1002/anie.201901575, (2019).

(88) Li, L.; Ma, D.-K.; Qi, F.; Chen, W.; Huang, S. Bi nanoparticles/Bi2O3 nanosheets with abundant grain boundaries for efficient electrocatalytic CO2 reduction. *Electrochim. Acta 298*, 580–586, DOI: 10.1016/j.electacta.2018.12.116, (2019).

(89) Hu, X.-M.; Hval, H. H.; Bjerglund, E. T.; Dalgaard, K. J.; Madsen, M. R.; Pohl, M.-M.; Welter, E.; Lamagni, P.; Buhl, K. B.; Bremholm, M.; Beller, M.; Pedersen, S. U.; Skrydstrup, T.; Daasbjerg, K. Selective CO_2 Reduction to CO in Water using Earth-Abundant Metal and Nitrogen-Doped Carbon Electrocatalysts. *ACS Catal. 8*, 6255–6264, DOI: 10.1021/acscatal.8b01022, (2018).

(90) Zhang, L.; Wu, N.; Zhang, J.; Hu, Y.; Wang, Z.; Zhuang, L.; Jin, X. Imidazolium

Ions with an Alcohol Substituent for Enhanced Electrocatalytic Reduction of CO_2. *ChemSusChem 10*, 4824–4828, DOI: 10.1002/cssc.201701673, (2017).

(91) Liu, K.; Wang, J.; Shi, M.; Yan, J.; Jiang, Q. Simultaneous Achieving of High Faradaic Efficiency and CO Partial Current Density for CO_2 Reduction via Robust, Noble-Metal-Free Zn Nanosheets with Favorable Adsorption Energy. *Adv. Energy Mater. 9*, 1900276, DOI: 10.1002/aenm.201900276, (2019).

(92) Zhu, W.; Zhang, L.; Yang, P.; Hu, C.; Dong, H.; Zhao, Z.-J.; Mu, R.; Gong, J. Formation of Enriched Vacancies for Enhanced CO_2 Electrocatalytic Reduction over AuCu Alloys. *ACS Energy Lett. 3*, 2144–2149, DOI: 10.1021/acsenergylett.8b01286, (2018).

(93) Sun, X.; Lu, L.; Zhu, Q.; Wu, C.; Yang, D.; Chen, C.; Han, B. MoP Nanoparticles Supported on Indium-Doped Porous Carbon: Outstanding Catalysts for Highly Efficient CO_2 Electroreduction. *Angew. Chem. Int. Ed. 57*, 2427–2431, DOI: 10.1002/anie.201712221, (2018).

(94) Ghausi, M. A.; Xie, J.; Li, Q.; Wang, X.; Yang, R.; Wu, M.; Wang, Y.; Dai, L. CO_2 Overall Splitting by a Bifunctional Metal-Free Electrocatalyst. *Angew. Chem. Int. Ed. 57*, 13135–13139, DOI: 10.1002/anie.201807571, (2018).

(95) Verma, S.; Hamasaki, Y.; Kim, C.; Huang, W.; Lu, S.; Jhong, H.-R. M.; Gewirth, A. A.; Fujigaya, T.; Nakashima, N.; Kenis, P. J. A. Insights into the Low Overpotential Electroreduction of CO_2 to CO on a Supported Gold Catalyst in an Alkaline Flow Electrolyzer. *ACS Energy Lett. 3*, 193–198, DOI: 10.1021/acsenergylett.7b01096, (2018).

(96) Hsieh, Y.-C.; Betancourt, L. E.; Senanayake, S. D.; Hu, E.; Zhang, Y.; Xu, W.; Polyansky, D. E. Modification of CO_2 Reduction Activity of Nanostructured Silver

Electrocatalysts by Surface Halide Anions. *ACS Appl. Energy Mater. 2*, 102–109, DOI: 10.1021/acsaem.8b01692, (2019).

(97) Ma, M.; Djanashvili, K.; Smith, W. A. Selective electrochemical reduction of CO_2 to CO on CuO-derived Cu nanowires. *Phys. Chem. Chem. Phys. 17*, 20861–20867, DOI: 10.1039/C5CP03559G, (2015).

(98) Gu, J.; Hsu, C.-S.; Bai, L.; Chen, H. M.; Hu, X. Atomically dispersed Fe^{3+} sites catalyze efficient CO_2 electroreduction to CO. *Science 364*, 1091–1094, DOI: 10.1126/science.aaw7515, (2019).

(99) Miao, C.-C.; Yuan, G.-Q. Morphology-Controlled Bi_2O_3 Nanoparticles as Catalysts for Selective Electrochemical Reduction of CO_2 to Formate. *ChemElectroChem 5*, 3741–3747, DOI: 10.1002/celc.201801036, (2018).

(100) He, R.; Zhang, A.; Ding, Y.; Kong, T.; Xiao, Q.; Li, H.; Liu, Y.; Zeng, J. Achieving the Widest Range of Syngas Proportions at High Current Density over Cadmium Sulfoselenide Nanorods in CO_2 Electroreduction. *Adv. Mater. 30*, 1705872, DOI: 10.1002/adma.201705872, (2018).

(101) Todoroki, N.; Tei, H.; Tsurumaki, H.; Miyakawa, T.; Inoue, T.; Wadayama, T. Surface Atomic Arrangement Dependence of Electrochemical CO_2 Reduction on Gold: Online Electrochemical Mass Spectrometric Study on Low-Index Au(*hkl*) Surfaces. *ACS Catal. 9*, 1383–1388, DOI: 10.1021/acscatal.8b04852, (2019).

(102) Huang, J.; Guo, X.; Wei, Y.; Hu, Q.; Yu, X.; Wang, L. A renewable, flexible and robust single layer nitrogen-doped graphene coating Sn foil for boosting formate production from electrocatalytic CO2 reduction. *J. CO2 Util. 33*, 166–170, DOI: 10.1016/j.jcou.2019.05.026, (2019).

(103) He, S.; Ni, F.; Ji, Y.; Wang, L.; Wen, Y.; Bai, H.; Liu, G.; Zhang, Y.; Li, Y.; Zhang, B.;

Peng, H. The p-Orbital Delocalization of Main-Group Metals to Boost CO_2 Electroreduction. *Angew. Chem. 130*, 16346–16351, DOI: 10.1002/ange.201810538, (2018).

(104) Zhao, C. et al. Solid-Diffusion Synthesis of Single-Atom Catalysts Directly from Bulk Metal for Efficient CO2 Reduction. *Joule 3*, 584–594, DOI: 10.1016/j.joule.2018.11.008, (2019).

(105) Vasileff, A.; Zheng, Y.; Qiao, S. Z. Carbon Solving Carbon's Problems: Recent Progress of Nanostructured Carbon-Based Catalysts for the Electrochemical Reduction of CO_2. *Adv. Energy Mater. 7*, 1700759, DOI: 10.1002/aenm.201700759, (2017).

(106) Xia, Z.; Freeman, M.; Zhang, D.; Yang, B.; Lei, L.; Li, Z.; Hou, Y. Highly Selective Electrochemical Conversion of CO_2 to HCOOH on Dendritic Indium Foams. *ChemElectroChem 5*, 253–259, DOI: 10.1002/celc.201700935, (2018).

(107) Zhu, M.; Chen, J.; Guo, R.; Xu, J.; Fang, X.; Han, Y.-F. Cobalt phthalocyanine coordinated to pyridine-functionalized carbon nanotubes with enhanced CO2 electroreduction. *Appl. Catal. B 251*, 112–118, DOI: 10.1016/j.apcatb.2019.03.047, (2019).

(108) Liu, S.; Yang, H. B.; Hung, S.; Ding, J.; Cai, W.; Liu, L.; Gao, J.; Li, X.; Ren, X.; Kuang, Z.; Huang, Y.; Zhang, T.; Liu, B. Elucidating the Electrocatalytic CO_2 Reduction Reaction over a Model Single-Atom Nickel Catalyst. *Angew. Chem. Int. Ed. 59*, 798–803, DOI: 10.1002/anie.201911995, (2020).

(109) Zhao, Y.; Liang, J.; Wang, C.; Ma, J.; Wallace, G. G. Tunable and Efficient Tin Modified Nitrogen-Doped Carbon Nanofibers for Electrochemical Reduction of Aqueous Carbon Dioxide. *Adv. Energy Mater. 8*, 1702524, DOI: 10.1002/aenm.201702524, (2018).

(110) Chang, X.; Wang, T.; Zhao, Z.; Yang, P.; Greeley, J.; Mu, R.; Zhang, G.; Gong, Z.; Luo, Z.; Chen, J.; Cui, Y.; Ozin, G. A.; Gong, J. Tuning Cu/Cu_2O Interfaces for the

Reduction of Carbon Dioxide to Methanol in Aqueous Solutions. *Angew. Chem. 130*, 15641–15645, DOI: 10.1002/ange.201805256, (2018).

(111) Song, J. T.; Ryoo, H.; Cho, M.; Kim, J.; Kim, J.-G.; Chung, S.-Y.; Oh, J. Nanoporous Au Thin Films on Si Photoelectrodes for Selective and Efficient Photoelectrochemical CO_2 Reduction. *Adv. Energy Mater. 7*, 1601103, DOI: 10.1002/aenm.201601103, (2017).

(112) Liu, X.; Schlexer, P.; Xiao, J.; Ji, Y.; Wang, L.; Sandberg, R. B.; Tang, M.; Brown, K. S.; Peng, H.; Ringe, S.; Hahn, C.; Jaramillo, T. F.; Nørskov, J. K.; Chan, K. pH effects on the electrochemical reduction of CO(2) towards C2 products on stepped copper. *Nat. Commun. 10*, 32, DOI: 10.1038/s41467-018-07970-9, (2019).

(113) Huang, P.; Cheng, M.; Zhang, H.; Zuo, M.; Xiao, C.; Xie, Y. Single Mo atom realized enhanced CO2 electro-reduction into formate on N-doped graphene. *Nano Energy 61*, 428–434, DOI: 10.1016/j.nanoen.2019.05.003, (2019).

(114) Sun, K.; Wu, L.; Qin, W.; Zhou, J.; Hu, Y.; Jiang, Z.; Shen, B.; Wang, Z. Enhanced electrochemical reduction of CO_2 to CO on Ag electrocatalysts with increased unoccupied density of states. *J. Mater. Chem. A 4*, 12616–12623, DOI: 10.1039/C6TA04325A, (2016).

(115) Wu, H.; Zeng, M.; Zhu, X.; Tian, C.; Mei, B.; Song, Y.; Du, X.-L.; Jiang, Z.; He, L.; Xia, C.; Dai, S. Defect Engineering in Polymeric Cobalt Phthalocyanine Networks for Enhanced Electrochemical CO_2 Reduction. *ChemElectroChem 5*, 2717–2721, DOI: 10.1002/celc.201800806, (2018).

(116) Yang, F.; Song, P.; Liu, X.; Mei, B.; Xing, W.; Jiang, Z.; Gu, L.; Xu, W. Highly Efficient CO_2 Electroreduction on ZnN_4-based Single-Atom Catalyst. *Angew. Chem. Int. Ed. 57*, 12303–12307, DOI: 10.1002/anie.201805871, (2018).

(117) Choi, J.; Benedetti, T. M.; Jalili, R.; Walker, A.; Wallace, G. G.; Officer, D. L. High Performance Fe Porphyrin/Ionic Liquid Co-catalyst for Electrochemical CO_2 Reduction. *Chem. Eur. J. 22*, 14158–14161, DOI: 10.1002/chem.201603359, (2016).

(118) Fan, Q.; Hou, P.; Choi, C.; Wu, T.; Hong, S.; Li, F.; Soo, Y.; Kang, P.; Jung, Y.; Sun, Z. Activation of Ni Particles into Single Ni–N Atoms for Efficient Electrochemical Reduction of CO_2. *Adv. Energy Mater. 10*, 1903068, DOI: 10.1002/aenm.201903068, (2020).

(119) Zhang, L.; Wang, Z.; Mehio, N.; Jin, X.; Dai, S. Thickness- and Particle-Size-Dependent Electrochemical Reduction of Carbon Dioxide on Thin-Layer Porous Silver Electrodes. *ChemSusChem 9*, 428–432, DOI: 10.1002/cssc.201501637, (2016).

(120) Han, J.; An, P.; Liu, S.; Zhang, X.; Wang, D.; Yuan, Y.; Guo, J.; Qiu, X.; Hou, K.; Shi, L.; Zhang, Y.; Zhao, S.; Long, C.; Tang, Z. Reordering d Orbital Energies of Single-Site Catalysts for CO_2 Electroreduction. *Angew. Chem. Int. Ed. 58*, 12711–12716, DOI: 10.1002/anie.201907399, (2019).

(121) Hu, H.; Gui, L.; Zhou, W.; Sun, J.; Xu, J.; Wang, Q.; He, B.; Zhao, L. Partially reduced Sn/SnO2 porous hollow fiber: A highly selective, efficient and robust electrocatalyst towards carbon dioxide reduction. *Electrochim. Acta 285*, 70–77, DOI: 10.1016/j.electacta.2018.08.002, (2018).

(122) Koh, J. H.; Jeon, H. S.; Jee, M. S.; Nursanto, E. B.; Lee, H.; Hwang, Y. J.; Min, B. K. Oxygen Plasma Induced Hierarchically Structured Gold Electrocatalyst for Selective Reduction of Carbon Dioxide to Carbon Monoxide. *J. Phys. Chem. C 119*, 883–889, DOI: 10.1021/jp509967m, (2015).

(123) Shen, Q.; Chen, Z.; Huang, X.; Liu, M.; Zhao, G. High-Yield and Selective Photoelectrocatalytic Reduction of CO_2 to Formate by Metallic Copper Decorated Co_3O_4

Nanotube Arrays. *Env. Sci. Tech. 49*, 5828–5835, DOI: 10.1021/acs.est.5b00066, (2015).

(124) Morlanés, N.; Takanabe, K.; Rodionov, V. Simultaneous Reduction of CO_2 and Splitting of H_2O by a Single Immobilized Cobalt Phthalocyanine Electrocatalyst. *ACS Catal. 6*, 3092–3095, DOI: 10.1021/acscatal.6b00543, (2016).

(125) Liu, M. et al. Quantum-Dot-Derived Catalysts for CO2 Reduction Reaction. *Joule 3*, 1703–1718, DOI: 10.1016/j.joule.2019.05.010, (2019).

(126) Xiang, H.; Rasul, S.; Scott, K.; Portoles, J.; Cumpson, P.; Yu, E. H. Enhanced selectivity of carbonaceous products from electrochemical reduction of CO2 in aqueous media. *J. CO2 Util. 30*, 214–221, DOI: 10.1016/j.jcou.2019.02.007, (2019).

(127) Han, H.; Park, S.; Jang, D.; Lee, S.; Kim, W. B. Electrochemical Reduction of CO_2 to CO by N,S Dual-Doped Carbon Nanoweb Catalysts. *ChemSusChem 13*, 539–547, DOI: 10.1002/cssc.201903117, (2020).

(128) Jeong, H.; Balamurugan, M.; Choutipalli, V. S. K.; Jo, J.; Baik, H.; Subramanian, V.; Kim, M.; Sim, U.; Nam, K. T. Tris(2-benzimidazolylmethyl)amine-Directed Synthesis of Single-Atom Nickel Catalysts for Electrochemical CO Production from CO_2. *Chem. Eur. J. 24*, 18444–18454, DOI: 10.1002/chem.201803615, (2018).

(129) Hu, X.; Yang, H.; Guo, M.; Gao, M.; Zhang, E.; Tian, H.; Liang, Z.; Liu, X. Synthesis and Characterization of (Cu, S) Co-doped SnO_2 for Electrocatalytic Reduction of CO_2 to Formate at Low Overpotential. *ChemElectroChem 5*, 1330–1335, DOI: 10.1002/celc.201800104, (2018).

(130) Zhang, X.; Sun, X.; Guo, S.-X.; Bond, A. M.; Zhang, J. Formation of lattice-dislocated bismuth nanowires on copper foam for enhanced electrocatalytic CO_2 reduction at low overpotential. *Energy & Environmental Science 12*, 1334–1340, DOI: 10.1039/C9EE00018F, (2019).

(131) Zheng, T.; Jiang, K.; Ta, N.; Hu, Y.; Zeng, J.; Liu, J.; Wang, H. Large-Scale and Highly Selective CO2 Electrocatalytic Reduction on Nickel Single-Atom Catalyst. *Joule 3*, 265–278, DOI: 10.1016/j.joule.2018.10.015, (2019).

(132) Todoroki, N.; Tei, H.; Miyakawa, T.; Tsurumaki, H.; Wadayama, T. Electrochemical CO $_2$ Reduction on Bimetallic Surface Alloys: Enhanced Selectivity to CO for Co/Au(110) and to H $_2$ for Sn/Au(110). *ChemElectroChem 6*, 3101–3107, DOI: 10.1002/celc.201900725, (2019).

(133) Gao, S.; Sun, Z.; Liu, W.; Jiao, X.; Zu, X.; Hu, Q.; Sun, Y.; Yao, T.; Zhang, W.; Wei, S.; Xie, Y. Atomic layer confined vacancies for atomic-level insights into carbon dioxide electroreduction. *Nat. Commun. 8*, 14503, DOI: 10.1038/ncomms14503, (2017).

(134) Yang, H.-J.; Dong, J.; Hong, Y.-H.; Lin, W.-F.; Zhou, Z.-Y.; Sun, S.-G. Comparative investigation of CO2 and oxygen reduction on Fe/N/C catalysts. *Electrochem. Commun. 97*, 82–86, DOI: 10.1016/j.elecom.2018.10.014, (2018).

(135) Wang, J.; Gan, L.; Zhang, Q.; Reddu, V.; Peng, Y.; Liu, Z.; Xia, X.; Wang, C.; Wang, X. A Water-Soluble Cu Complex as Molecular Catalyst for Electrocatalytic CO $_2$ Reduction on Graphene-Based Electrodes. *Adv. Energy Mater. 9*, 1803151, DOI: 10.1002/aenm.201803151, (2019).

(136) Wang, X.; Xiao, W.; Zhang, J.; Wang, Z.; Jin, X. Nanoporous Ag-Sn derived from codeposited AgCl-SnO2 for the electrocatalytic reduction of CO2 with high formate selectivity. *Electrochem. Commun. 102*, 52–56, DOI: 10.1016/j.elecom.2019.03.017, (2019).

(137) Zhang, Q.; Zhang, Y.; Mao, J.; Liu, J.; Zhou, Y.; Guay, D.; Qiao, J. Electrochemical Reduction of CO $_2$ by SnO $_x$ Nanosheets Anchored on Multiwalled Carbon

Nanotubes with Tunable Functional Groups. *ChemSusChem 12*, 1443–1450, DOI: 10.1002/cssc.201802725, (2019).

(138) Rabiee, A.; Nematollahi, D. Electrochemical reduction of CO2 to formate ion using nanocubic mesoporous In(OH)3/carbon black system. *Mater. Chem. Phys. 193*, 109–116, DOI: 10.1016/j.matchemphys.2017.02.016, (2017).

(139) Lv, K.; Teng, C.; Shi, M.; Yuan, Y.; Zhu, Y.; Wang, J.; Kong, Z.; Lu, X.; Zhu, Y. Hydrophobic and Electronic Properties of the E-MoS $_2$ Nanosheets Induced by FAS for the CO $_2$ Electroreduction to Syngas with a Wide Range of CO/H $_2$ Ratios. *Adv. Funct. Mater. 28*, 1802339, DOI: 10.1002/adfm.201802339, (2018).

(140) Min, S.; Yang, X.; Lu, A.-Y.; Tseng, C.-C.; Hedhili, M. N.; Li, L.-J.; Huang, K.-W. Low overpotential and high current CO2 reduction with surface reconstructed Cu foam electrodes. *Nano Energy 27*, 121–129, DOI: 10.1016/j.nanoen.2016.06.043, (2016).

(141) Lei, F.; Liu, W.; Sun, Y.; Xu, J.; Liu, K.; Liang, L.; Yao, T.; Pan, B.; Wei, S.; Xie, Y. Metallic tin quantum sheets confined in graphene toward high-efficiency carbon dioxide electroreduction. *Nat. Commun. 7*, 12697, DOI: 10.1038/ncomms12697, (2016).

(142) Zhang, M.; Wu, T.-S.; Hong, S.; Fan, Q.; Soo, Y.-L.; Masa, J.; Qiu, J.; Sun, Z. Efficient Electrochemical Reduction of CO $_2$ by Ni–N Catalysts with Tunable Performance. *ACS Sust. Chem. Eng. 7*, 15030–15035, DOI: 10.1021/acssuschemeng.9b03502, (2019).

(143) Chen, Z.; Mou, K.; Yao, S.; Liu, L. Highly selective electrochemical reduction of CO $_2$ to formate on metal-free nitrogen-doped PC61BM. *J. Mater. Chem. A 6*, 11236–11243, DOI: 10.1039/C8TA03328E, (2018).

(144) Pan, F.; Liang, A.; Duan, Y.; Liu, Q.; Zhang, J.; Li, Y. Self-growth-templating synthesis of 3D N,P,Co-doped mesoporous carbon frameworks for efficient bifunctional

oxygen and carbon dioxide electroreduction. *J. Mater. Chem. A 5*, 13104–13111, DOI: 10.1039/C7TA03005C, (2017).

(145) Zhou, J.; Yuan, K.; Zhou, L.; Guo, Y.; Luo, M.; Guo, X.; Meng, Q.; Zhang, Y. Boosting Electrochemical Reduction of CO_2 at a Low Overpotential by Amorphous Ag-Bi-S-O Decorated Bi^0 Nanocrystals. *Angew. Chem. Int. Ed. 58*, 14197–14201, DOI: 10.1002/anie.201908735, (2019).

(146) Kou, W.; Zhang, Y.; Dong, J.; Mu, C.; Xu, L. Nickel–Nitrogen-Doped Three-Dimensional Ordered Macro-/Mesoporous Carbon as an Efficient Electrocatalyst for CO_2 Reduction to CO. *ACS Appl. Energy Mater. 3*, 1875–1882, DOI: 10.1021/acsaem.9b02324, (2020).

(147) He, S.; Ji, D.; Zhang, J.; Novello, P.; Li, X.; Zhang, Q.; Zhang, X.; Liu, J. Understanding the Origin of Selective Reduction of CO_2 to CO on Single-Atom Nickel Catalyst. *J. Phys. Chem. B 124*, 511–518, DOI: 10.1021/acs.jpcb.9b09730, (2020).

(148) Wuttig, A.; Yaguchi, M.; Motobayashi, K.; Osawa, M.; Surendranath, Y. Inhibited proton transfer enhances Au-catalyzed CO2-to-fuels selectivity. *Proc. Natl. Acad. Sci. 113*, E4585–E4593, (2016).

(149) Dunwell, M.; Luc, W.; Yan, Y.; Jiao, F.; Xu, B. Understanding surface-mediated electrochemical reactions: CO2 reduction and beyond. *ACS Catal. 8*, 8121–8129, (2018).

(150) Williams, K.; Corbin, N.; Zeng, J.; Lazouski, N.; Yang, D.-T.; Manthiram, K. Protecting effect of mass transport during electrochemical reduction of oxygenated carbon dioxide feedstocks. *Sustain. Energy Fuels 3*, 1225–1232, (2019).
