# Supplementary material for: Bayesian data analysis reveals no preference for cardinal Tafel slopes in CO2 reduction electrocatalysis
Source: Nat Commun. 2021 Jan 29;12:703. doi: 10.1038/s41467-021-20924-y (PMC7846806; doi:10.1038/s41467-021-20924-y)
Supplement: Supplementary file 2 — Peer Review File [file 41467_2021_20924_MOESM2_ESM.pdf]

Editorial Note: This manuscript has been previously reviewed at another journal that is not operating a transparent peer review scheme. This document only contains reviewer comments and rebuttal letters for versions considered at *Nature Communications.*

Reviewer #1 (Remarks to the Author):

The authors have made great efforts to address the reviewers' concerns and the quality of this work is further improved. In my side, I have no further question or suggestion. I recommend publication of this piece of work in Nature Communications to raise awareness of properly doing Tafel analysis in electrocatalysis community.

Reviewer #2 (Remarks to the Author):

The authors have provided a thoughtful and detailed response to all of the reviews, with revisions that have strengthened the paper. Clearly, all 4 reviewers both recognized the value in this study, as well as the complexity involved in using a simple Tafel kinetic/transport model, as do the authors. Presuming the reviewers are indicative of the readers of the paper, the consistency in reviews makes it clear the level of discussion in the paper is quite reasonable and well targetted. The authors have also improved in presenting the use of multiple-reaction models and further discussions of other complexities to make these apparent to all readers. Frankly, I am disappointed that this will not be published in Nature Catalysis, as a targetted catalysis audience is a better place for this work and I remain supportive that it reaches the qualtity for publication in that journal. Whether Nature Catalysis or Nature Communications, I recommend publication of the revised manuscript.

Reviewer comments in black
Author responses in blue

Reviewer #1 (Remarks to the Author):

The authors have made great efforts to address the reviewers' concerns and the quality of this work is further improved. In my side, I have no further question or suggestion. I recommend publication of this piece of work in Nature Communications to raise awareness of properly doing Tafel analysis in electrocatalysis community.

No action required here.

Reviewer #2 (Remarks to the Author):

The authors have provided a thoughtful and detailed response to all of the reviews, with revisions that have strengthened the paper. Clearly, all 4 reviewers both recognized the value in this study, as well as the complexity involved in using a simple Tafel kinetic/transport model, as do the authors. Presuming the reviewers are indicative of the readers of the paper, the consistency in reviews makes it clear the level of discussion in the paper is quite reasonable and well targetted. The authors have also improved in presenting the use of multiple-reaction models and further discussions of other complexities to make these apparent to all readers. Frankly, I am disappointed that this will not be published in Nature Catalysis, as a targetted catalysis audience is a better place for this work and I remain supportive that it reaches the qualtity for publication in that journal. Whether Nature Catalysis or Nature Communications, I recommend publication of the revised manuscript.

No action required here.
